# Multiplex peptide microarray profiling of antibody reactivity against neglected tropical diseases derived B-cell epitopes for serodiagnosis in Zimbabwe

**DOI:** 10.1371/journal.pone.0271916

**Published:** 2022-07-22

**Authors:** Arthur Vengesai, Thajasvarie Naicker, Herald Midzi, Maritha Kasambala, Tariro L. Mduluza-Jokonya, Simbarashe Rusakaniko, Francisca Mutapi, Takafira Mduluza

**Affiliations:** 1 Department of Biochemistry, Faculty of Medicine, Midlands State University, Gweru, Zimbabwe; 2 Department of Optics and Imaging, College of Health Sciences, University of KwaZulu-Natal, Durban, South Africa; 3 Department of Biotechnology and Biochemistry, Faculty of Science, University of Zimbabwe, Harare, Zimbabwe; 4 Department of Biological Sciences and Ecology, Faculty of Science, University of Zimbabwe, Harare, Zimbabwe; 5 Family Medicine, Global and Public Health Unit, Faculty of Medicine and Health Sciences, University of Zimbabwe, Harare, Zimbabwe; 6 Institute for Immunology and Infection Research and Centre for Immunity, Infection and Evolution, School of Biological Sciences, University of Edinburgh, Ashworth Laboratories, Edinburgh, United Kingdom; Public Library of Science, UNITED KINGDOM

## Abstract

**Introduction:**

Peptides (B-cell epitopes) have broad applications in disease diagnosis and surveillance of pathogen exposure. In this framework, we present a pilot study to design and produce a peptide microarray for the integrated surveillance of neglected tropical diseases. The peptide microarray was evaluated against peptides derived from *Ascaris lumbricoides*, *Necator americanus*, *Schistosoma haematobium*, *Schistosoma mansoni*, *Trichuris trichiura*, *Bacillus anthracis*, *Mycobacterium leprae*, *Wuchereria bancrofti*, *Rabies lyssavirus*, *Chlamydia trachomatis* and *Trypanosoma brucei*.

**Methods:**

*S*. *haematobium* was diagnosed using the urine filtration technique. *S*. *mansoni*, *A*. *lumbricoides*, *N*. *americanus* and *T*. *trichiura* were diagnosed using the Kato Katz and formal ether concentration techniques. Immunogenic peptides were retrieved from the Tackling Infection to Benefit Africa infectious diseases epitope microarray. Further peptides were predicted using ABCpred. IgG and IgM reactivity against the derived peptides were evaluated using peptide microarray multiplex immunoassays. Positive response was defined as fluorescence intensity ≥ 500 fluorescence units. Immunodominant peptides were identified using color-coded heat maps and bar graphs reflecting the obtained fluorescence signal intensities. Receiver Operating Characteristic analysis and Mann-Whitney-U test were performed to determine the diagnostic validity of the peptides.

**Results:**

Species-specific responses with at least one peptide derived from each NTD pathogen were observed. The reactive peptides included; for *S*. *haematobium*, XP_035588858.1-206-220 and XP_035588858.1-206-220 immunodominant for IgG and IgM respectively, for *S*. *mansoni*, P20287.1-58-72 immunodominant for both antibodies and for *T*. *trichiura*, CDW52482.1-326-340 immunodominant for IgG and CDW57769.1-2017-2031 and CDW57769.1-1518-1532 immunodominant for IgM. According to ROC analysis most of the peptides selected were inaccurate; with AUC < 0.5. Some peptides had AUC values ranging from 0.5 to 0.5875 for both IgM and IgG suggesting no discrimination.

**Conclusion:**

Multiplex peptide microarrays are a valuable tool for integrated NTDs surveillance and for screening parasites exposure in endemic areas. Species sero-reactivity observed in the study maybe indicative of exposure to the different NTDs parasites. However, although peptides with the least cross reactivity were selected there is need to validate the sero-reactivity with recombinant antigens and immune-blotting techniques such as western blotting.

## Introduction

Neglected Tropical Diseases (NTDs) are a group of 20 bacterial, parasitic and viral chronic infectious diseases that affect over 1.7 billion people globally and are particularly endemic to the tropical and subtropical regions [1, 2]. They include, schistosomiasis (mainly caused by *Schistosoma haematobium*, *Schistosoma mansoni and Schistosoma japonicum*), soil-transmitted helminthiasis (STH) (commonly caused by *Ascaris lumbricoides*, *Necator americanus and Trichuris trichiura*), lymphatic filariasis (caused by *Wuchereria bancrofti)*, blinding trachoma (caused by *Chlamydia trachomatis*), leprosy (caused by *Mycobacterium leprae*) and Human African trypanosomiasis (caused *Trypanosoma brucei*) [1–4]. NTDs also include zoonotic diseases such as rabies (caused by *Rabies lyssavirus*) and anthrax (caused by *Bacillus anthracis*) [5].

NTDs are intimately related to poverty and they tend to cluster in the same poor populations [6, 7]. Zimbabwe which is in the Southern region of Africa is endemic to four of the most common NTDs; Schistosomiasis, STH, lymphatic filariasis and trachoma. In 2016 nearly 10 million Zimbabweans required preventative chemotherapy for at least 1 NTD [8]. NTDs mapping results showed that of the 63 districts in Zimbabwe, 56 are endemic for schistosomiasis, 47 are endemic for STH and 39 for lymphatic filariasis [9, 10].

Engels and Savioli suggested that there is need for an integrated approach to eradicate NTDs [7]. Advances in serological multiplex immunoassays have created enormous potential for large-scale, integrated NTDs surveillance [11]. The parallel detection of antibodies has a wide range of potential applications in the diagnosis and surveillance of NTDs as well as in epitope mapping studies, therapeutics and vaccines development [12, 13]. Peptide microarrays provide rapid and high-throughput immunoassay platforms for the simultaneous identification of B-cell epitopes derived from different NTDs parasites. B-cell epitopes have broad applications in the development of peptide based vaccines, in NTDs diagnosis and surveillance of pathogen exposure [14].

With this background, we present a pilot study to design and produce a peptide microarray in a laser-printer based approach and validate the microarray using human serum and plasma samples from three rural districts in Zimbabwe. The peptide microarray carried a panel of fifty-one, 9–18 amino acids B-cell epitopes derived from *A*. *lumbricoides*, *N*. *americanus*, *S*. *haematobium*, *S*. *mansoni*, *T*. *trichiura*, *B*. *anthracis*, *M*. *Leprae*, *W*. *bancrofti*, *Rabies lyssavirus*, *C*. *trachomatis* and *T*. *brucei*. Peptide microarray immunoassays were also established to evaluate the diagnostic performance of peptides derived from *A*. *lumbricoides*, *N*. *americanus*, *S*. *haematobium*, *S*. *mansoni* and *T*. *trichiura*.

## Materials and methods

### Ethical approval

Ethical approval was obtained from the Medical Research Council of Zimbabwe (MCRZ/A/2571 and MRCZ/A/2443). Permission to conduct the study in the districts was granted by the Provincial Medical Directors, District Medical Officers, councillors, and village headmen. Participants provided written consent prior to recruitment. Parents and guardians provided written consent for children after the children had given their assent. Prior to recruitment study objectives were explained to the participants, parents and guardians in both Shona and English languages.

### Study area and population

Villagers of all age groups living in NTDs endemic areas were purposively selected for the cross-sectional study. The villagers were from Shamva, Murewa and Makoni rural districts in Zimbabwe. Shamva and Murewa rural districts are located in Mashonaland Central province (31°40′0” E longitude and 17°10′0” S latitude) and Mashonaland East province (17°38′49″S latitude and 31°46′39″E longitude) in northeastern Zimbabwe respectively, whilst Makoni district is located in Manicaland province in eastern Zimbabwe (18°32’09.2"S latitude and 32°07’18.9"E longitude). The prevalence’s of *S*. *haematobium*, *S*. *mansoni*, *T*. *trichiura*, *N*. *americanus* and *A*. *lumbricoides* in the study provinces presented in **Table 1** were reported by Midzi *et al*., 2014 [9]. To be included in the study population villagers were required to provide three urine samples collected on consecutive days, one stool and one blood sample.

**Table 1:** Prevalence of *S*. *haematobium*, *S*. *mansoni*, *T*. *trichiura*, *N*. *americanus* and *A*. *lumbricoides in* Mashonaland Central, Mashonaland East and Manicaland in 2011.

Provinces	S. haematobium	S. mansoni	*N*. *americanus*	*A*. *lumbricoides*	*T*. *trichiura*
Mashonaland central	26.1%	20.4%	0.6%	1.0%	0.4%
Mashonaland East	28.1%	6.4%	1.1%	17.8%	0.2%
Manicaland	12.8%	14.3%	2.9%	1,9%	0.4%

### Parasitological examination

Urine and stool specimens were collected between 10:00 am and 14:00 pm for optimal egg passage necessary for diagnosis of schistosomiasis and STH. The samples were placed in wooden boxes away from sunlight until they were processed and examined. *S*. *haematobium* was diagnosed by the microscopic examination of urine for parasites eggs using the urine filtration technique. The technique was repeated for three consecutive days in order to avoid misdiagnosis due to day-to-day variation in egg excretion [15].

Stool samples were examined for the ova of *T*. *trichiura*, *N*. *americanus*, *A*. *lumbricoides* and *S*. *mansoni* using the Kato-Katz technique and the formal ether concentration technique [16, 17]. Participants were classified as infected if at least one parasitic egg was detected. Participants who tested positive for schistosomiasis and STH were referred to the nearest health centres for treatment. Healthy non-infected individuals from the NTDs endemic areas without parasite infections were considered as the negative control group. It is noteworthy that no parasitology diagnosis was conducted for *B*. *anthracis*, *M*. *Leprae*, *W*. *bancrofti*, *R*. *lyssavirus*, *C*. *trachomatis* and *T*. *brucei*.

### Peptide selection

The immunogenic peptides (B-cell epitopes) were retrieved from the PEPperPRINT and TIBA infectious disease epitope microarray databases. For novel peptides, a literature search for pathogen proteins was conducted in PubMed. The search focused on proteins found on the surface of the pathogens and secretory or excretory proteins. Protein sequences were then obtained either from NCBI (https://www.ncbi.nlm.nih.gov/) or Uniprot (https://www.uniprot.org/) protein databases. The prediction of linear B-cell epitopes on selected protein sequences was done using a bioinformatics tool ABCpred [14]. The NCBI Protein BLAST (https://blast.ncbi.nlm.nih.gov/Blast.cgi select Protein BLAST) bioinformatics tool was then used to select the peptides with the least cross-reactive. Predicted peptides with the ABCpred highest rank and with the least cross-reactivity with peptides from other human pathogens or proteins were selected for the study.

### Peptide microarray design and layout

**Fig 1:**
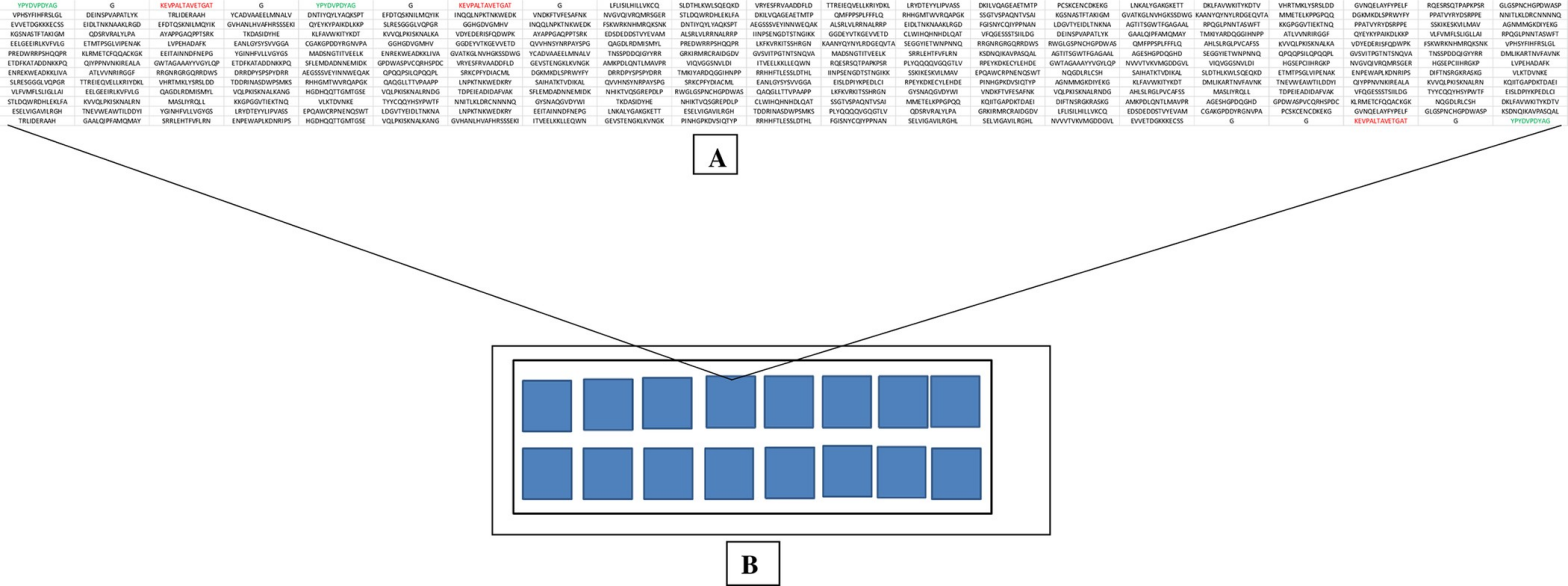
Peptide microarray chip design and layout. (**A**) Microarray peptide content, in black are 130 selected peptides printed with random distribution in duplicate to give 260 spots and in green and red are 3 HA and 3 Polio internal control spots. (**B**) Peptide microarray layout with 16 identical array copies (subarrays).

The peptide microarray was customer designed to include three to five 9aa-18aa peptides derived from each pathogen and was generated in a laser-printer based approach by PEPperPRINT GmbH (Heidelberg, Germany) (https://www.pepperprint.com/). The peptide microarray contained 16 identical sub-arrays (copies) with 260 peptide positions on each sub-array. Fifty-one duplicate NTDs peptides (details given in **Table 2**) were printed with random distribution across each sub-array. Each sub-array was framed by flag anti-polio (KEVPALTAVETGAT, 3 spots) and flag anti-HA (hemagglutinin glycoprotein of influenza virus) (YPYDVPDYAG, 3 spots) as a quality control measurement. Each sub-array was also framed by glycine spacers (G, 7 spots) as negative controls. See **Fig 1** for details on peptide microarray layout.

**Table 2:** TIBA infectious disease epitope microarray and ABCpred selected B-cell epitopes.

Peptide name	Linear sequance	Source organism	Source Molecule/ protein	Peptide prediction and selection
CAA60047.1**-553-568**	TMKIYARDQGGIHNPP	*Ascaris lumbricoides*	Cytoplasmic intermediate filament protein	ABC PRED
ACJ03763.1**-50-64**	TDPEIEADIDAFVAK	*Ascaris lumbricoides*	Ag2	TIBA infectious disease epitope microarray
ACJ03764.1**-3852-38-52**	KQIITGAPDKTDAEI	*Ascaris lumbricoides*	Ag1	TIBA infectious disease epitope microarray
AAD13652.1**-133-147**	RRHHFTLESSLDTHL	*Ascaris lumbricoides*	Aba-1 Allergen, Partial	TIBA infectious disease epitope microarray
AAD13652.1**-142-156**	SLDTHLKWLSQEQKD	*Ascaris lumbricoides*	Aba-1 Allergen Partial	TIBA infectious disease epitope microarray
WP_151277871.1**-342-358**	GVATKGLNVHGKSSDWG	*Bacillus anthracis*	anthrax toxin edema factor	TIBA infectious disease epitope microarray
WP_040119992.1**-736-358**	IINPSENGDTSTNGIKK	*Bacillus anthracis*	Chain A Anthrax Protective Antigen	TIBA infectious disease epitope microarray
WP_001022096.1**-35-44**	GGHGDVGMHV	*Bacillus anthracis*	Lethal Factor	TIBA infectious disease epitope microarray
WP_151277871.1**-307-316**	LVPEHADAFK	*Bacillus anthracis*	Edema Factor	TIBA infectious disease epitope microarray
P0C0Z7.2**-260-270**	ATLVVNRIRGGF	*Chlamydia trachomatis*	60 Kda Chaperonin (Protein Cpn60) (Groel Protein) (57 Kda Chlamydial Hypersensitivity Antigen) (Heat Shock Protein 60) (Hsp60)	TIBA infectious disease epitope microarray
P19542.1**-261-270**	TKDASIDYHE	*Chlamydia trachomatis*	Major Outer Membrane Porin, Serovar L1 Precursor (Momp)	TIBA infectious disease epitope microarray
AAO67542.1**-291-305**	LKFKVRKITSSHRGN	*Chlamydia trachomatis*	Outer Membrane Protein Porb	TIBA infectious disease epitope microarray
AAA23156.1**-381-390**	TRLIDERAAH	*Chlamydia trachomatis*	Major Outer Membrane Protein	TIBA infectious disease epitope microarray
AAA23156.1**-73-81**	VLKTDVNKE	*Chlamydia trachomatis*	Major Outer Membrane Protein	TIBA infectious disease epitope microarray
CAA43269.1**-311-325**	AMKPDLQNTLMAVPR	*Mycobacterium leprae*	Antigen 85-B Precursor	TIBA infectious disease epitope microarray
WP_010907696.1**-11-25**	DKILVQAGEAETMTP	*Mycobacterium leprae*	co-chaperone GroES	TIBA infectious disease epitope microarray
WP_010907618.1**-27-41**	LDGVTYEIDLTNKNA	*Mycobacterium leprae*	Protein Lsr2 Precursor	TIBA infectious disease epitope microarray
CAA37572.1**-10-24**	EIDLTNKNAAKLRGD	*Mycobacterium leprae*	Lsr2	TIBA infectious disease epitope microarray
WP_010907696.1**-1-15**	ETMTPSGLVIPENAK	*Mycobacterium leprae*	co-chaperone GroES	TIBA infectious disease epitope microarray
CAC00543.1**-123-135**	SRKCPFYDIACML	*Necator americanus*	Necepsin Ii	TIBA infectious disease epitope microarray
AAP41952.1**-180-192**	AGNMMGKDIYEKG	*Necator americanus*	Secreted Protein Asp-2	TIBA infectious disease epitope microarray
AAP41952.1**-194-206**	PCSKCENCDKEKG	*Necator americanus*	Secreted Protein Asp-2	TIBA infectious disease epitope microarray
AHC94315.1**-209-222**	DIFTNSRGKRASKG	*Rabies lyssavirus*	Glycoprotein G Precursor	ABC PRED
AAD10459.1**-312-325**	VPHSYFIHFRSLGL	*Rabies lyssavirus*	ribonucleoprotein (RNP)	ABC PRED
BAJ04981.1**-12-26**	QYEYKYPAIKDLKKP	*Rabies lyssavirus*	Nucleoprotein	TIBA infectious disease epitope microarray
QEJ74712.1**-99-113**	NVGVQIVRQMRSGER	*Rabies lyssavirus*	Phosphoprotein	TIBA infectious disease epitope microarray
XP_012799745.1**-16-30**	SFLEMDADNNEMIDK	*Schistosoma haematobium*	putative 22.6 kDa tegument antigen	TIBA infectious disease epitope microarray
XP_035587815.1**-269-283**	EISLDPIYKPEDLCI	*Schistosoma haematobium*	putative heat shock protein hsp16	TIBA infectious disease epitope microarray
XP_035588858.1**-206-220**	EDSDEDDSTVYEVAM	*Schistosoma haematobium*	putative cleavage and polyadenylation specificity factor	TIBA infectious disease epitope microarray
XP_012797374.1**-78-92**	NHIKTVQSGREPDLP	*Schistosoma haematobium*	Antigen Sm21.7	TIBA infectious disease epitope microarray
AAZ29530.1**-25-29**	PINHGPKDVSIQTYP	*Schistosoma haematobium*	Tegumental Protein Sh13	TIBA infectious disease epitope microarray
P09792.1**-29-43**	VDYEDERISFQDWPK	*Schistosoma mansoni*	Glutathione S-Transferase Class-Mu 28 Kda Isozyme	TIBA infectious disease epitope microarray
P20287.1**-58-72**	GEVSTENGKLKVNGK	*Schistosoma mansoni*	Glyceraldehyde-3-Phosphate Dehydrogenase (Gapdh) (Major Larval Surface Antigen) (P-37)	TIBA infectious disease epitope microarray
AAA29900.1**-145-159**	CGAKGPDDYRGNVPA	*Schistosoma mansoni*	23 Kda Integral Membrane Protein (Sm23)	TIBA infectious disease epitope microarray
P09841.3**-6-20**	LFLISILHILLVKCQ	*Schistosoma mansoni*	Hemoglobinase Precursor (Antigen Sm32)	TIBA infectious disease epitope microarray
AAA29903.1**-222-237**	KSDNQIKAVPASQAL	*Schistosoma mansoni*	Major Egg Antigen	TIBA infectious disease epitope microarray
CDW57769.1**-1518-1532**	VRYESFRVAADDFLD	*Trichuris trichiura*	Parp And Wgr And Ank 2 Domain Containing Protein	TIBA infectious disease epitope microarray
CDW57769.1**-659-673**	DMLIKARTNVFAVNK	*Trichuris trichiura*	Parp And Wgr And Ank 2 Domain Containing Protein	TIBA infectious disease epitope microarray
CDW57769.1**-834-848**	STLDQWRDHLEKLFA	*Trichuris trichiura*	Parp And Wgr And Ank 2 Domain Containing Protein	TIBA infectious disease epitope microarray
CDW52482.1**-326-340**	TNEVWEAWTILDDYI	*Trichuris trichiura*	Wap Domain Containing Protein Slpi-Like	TIBA infectious disease epitope microarray
CDW57769.1**-2017-2031**	RPEYKDKECYLEHDE	*Trichuris trichiura*	Parp And Wgr And Ank 2 Domain Containing Protein	TIBA infectious disease epitope microarray
APD74596.1**-96-110**	ENREKWEADKKLIVA	*Trypanosoma brucei*	Variant Surface Glycoprotein	TIBA infectious disease epitope microarray
CAC33895.1**-78-92**	ETDFKATADDNKKPQ	*Trypanosoma brucei*	VSG protein	TIBA infectious disease epitope microarray
XP_844815.1**-369-382**	SSKIKESKVILMAV	*Trypanosoma brucei gambiense*	64 kDa invariant surface glycoprotein	ABC PRED
CAC33895.1**-163-176**	LNKALYGAKGKETT	*Trypanosoma brucei gambiense*	variant surface glycoprotein LiTat 1.3	ABC PRED
XP_011774209.1**-132-145**	SAIHATKTVDIKAL	*Trypanosoma brucei gambiense DAL972*	mitogen-activated protein kinase 5	ABC PRED
AAC35355.1**-55-68**	EVVETDGKKKECSS	*Wuchereria bancrofti*	Abundant Larval Transcript-2 Protein	ABC PRED
AAC35355.1**-46-60**	GGDEYVTKGEVVETD	*Wuchereria bancrofti*	Abundant Larval Transcript-2 Protein	TIBA infectious disease epitope microarray
AAC35355.1**-77-91**	EPQAWCRPNENQSWT	*Wuchereria bancrofti*	Abundant Larval Transcript-2 Protein	TIBA infectious disease epitope microarray
AAC70783.1**-177-191**	FSKWRKNHMRQKSNK	*Wuchereria bancrofti*	Sxp Antigen	TIBA infectious disease epitope microarray
ADP24698.1**-29-43**	TDDRINASDWPSMKS	*Wuchereria bancrofti*	Glutathione S-Transferase pi class	TIBA infectious disease epitope microarray

The peptide name consisted of the protein/antigen accession number followed by the amino acids positions of the peptide in the protein.

### Peptide microarrays immunoassays

The immunoassays were performed in 3/16-well PEPperCHIP® Incubation Trays (PEPperPRINT GmbH, Germany), which allowed for the subdivision of the peptide microarray substrate glass slide into 16 separate incubation wells for each slide. The immunoassays consisted of two steps on the same microarray: the pre-incubation step for identifying false positive signals by binding of the fluorescently labelled secondary antibody followed by the main incubation with serum and the secondary antibodies.

Each step involved pre-swelling of the peptide microarrays with washing buffer (PBS, pH 7.4 with 0.05% Tween 20) for 10 minutes, followed by incubation with blocking buffer (Rockland blocking buffer MB-070) for 30 minutes. Initially the peptide microarrays were incubated with secondary antibodies [Goat anti-human IgG (Fc) DyLight680 (0.1 μg/ml) and goat anti-human IgM (μ chain) DyLight800 (0.2 μg/ml)] and control antibodies [Mouse monoclonal anti-HA DyLight800 (0.5 μg/ml)] diluted in incubation buffer (washing buffer with 10% blocking buffer) at room temperature for 45 minutes. In the main step the peptide microarrays were incubated with serum or plasma diluted 1:250 in incubation buffer for 16 h at 4°C and 140 rpm orbital shaking followed by incubation with the secondary antibodies. After each incubation step the peptide microarrays were washed three times with washing buffer for 10 seconds. The peptide microarrays were scanned using an LI-COR Odyssey Imaging System; scanning offset 0.65 mm, resolution 21 μm, scanning intensities of 7/7 (red = 680 nm / green = 800 nm). To ensure that all microarrays were responding correctly, all steps were repeated with the Cy3-conjugated anti-HA control antibody and Cy3-conjugated anti-polio control antibodies.

Prior data quantification, all the peptide microarray scans were visually assessed for possible assay or microarray artifacts which would affect the experimental outcome. If required, the assays with individual samples were repeated on new peptide microarrays. For technical reasons, read-out for repeated samples was carried out with an Innopsys InnoScan 710-IR microarray scanner with matching scanner settings.

### Image analysis and spot intensity quantification

Quantification of spot intensities was based on 16-bit gray scale tiff files. Microarray image analysis was done with PepSlide^®^ Analyzer (SICASYS Software GmbH; Heidelberg, Germany) and resulted in raw data CSV files for each sample (green = 800 nm = IgM staining, red = 680 nm = IgG staining). A PEPperPRINT software algorithm calculated averaged median foreground intensities (foreground-background signal) and spot-to-spot deviations of spot duplicates and assembled the outcome in summary files. For duplicate spots a maximum spot-to-spot deviation of 50% was tolerated, otherwise the corresponding intensity value was zeroed.

### Statistical analysis

Age was expressed as median and interquartile range (IQR). The data set (**S1 File**) used for statistical analysis of the peptide microarray results and for generation of all heat map presentations were based on fluorescence intensity. Duplicate fluorescence values were averaged in Microsoft excel 2013. Bar graphs were also drawn using Microsoft excel 2013.

Heat maps were generated online using Morpheus heat map widget (https://software.broadinstitute.org/morpheus/). Non-parametric statistical methods (Mann-Whitney-U test and nonparametric ROC analysis) were used for data analysis. Univariate comparisons of two independent groups were done using the Mann-Whitney-U test in SPSS 16.0. Area under the ROC curve (AUC) was measured for each peptide using STATA/MP16.0. P-values lower than 0.05 were considered statistically significant.

### Antibody reactivity and discrimination of infection by detection of immunodominant epitopes

A positive peptide response was defined as fluorescence intensity ≥ 500 Relative Fluorescent Units (RFU) for both IgG and IgM against each peptide [18]. Immunodominant peptides were identified using color-coded heat maps and bar graphs reflecting obtained fluorescence signal intensities. The ability of peptides to distinguish between the infected and uninfected groups was statistically evaluated using the Mann-Whitney-U test. Diagnostic accuracy of the peptides was evaluated by ROC curve analysis and AUC was calculated to assess the overall diagnostic performance of peptide (**S1 Table**). Results of antibody reactivity against the peptide microarray internal quality controls spots (KEVPALTAVETGAT, YPYDVPDYAG and G spots) has not been reported in the manuscript but are presented in **S2 Table**.

## Results

### Demography and parasitology data

The study consisted of 170 participants of which 49.1% were males, of all age groups with a median age of 11 (interquartile range 5.25–30.00). Madziwa had 74.7% (127) of the participants while the participants in Murewa and Makoni were 17.1% (29) and 8.2% (14) respectively. Among the villagers included in the study, 4 were infected with *A*. *lumbricoides*, 14 with *N*. *americanus*, 61 with *S*. *haematobium*, 44 with *S*. *mansoni* and 6 with *T*. *trichiura* whilst 60 were uninfected and were used as controls during analysis.

### Antibody reactivity and discrimination of *Ascaris lumbricoides* infection

*Ascaris lumbricoides* derived peptides were reactive with IgG except peptide CAA60047.1-553-568 (TMKIYARDQGGIHNPP) which did not react with neither *Ascaris lumbricoides* infected nor uninfected samples. Peptide ACJ03764.1-3852-38-52 (KQIITGAPDKTDAEI) gave the highest response with a fluorescence intensity of 13563.25 RFU with sera from the *Ascaris lumbricoides* infected group. For IgM, all *Ascaris lumbricoides* derived peptides were reactive with at least one sera from either the *Ascaris lumbricoides* infected or uninfected group. In contrast with IgG peptide ACJ03763.1-50-64 (TDPEIEADIDIAFVAK) gave the highest fluorescence intensity 2806.5 RFU. Looking at the heat maps for *Ascaris lumbricoides* derived peptide there was no immunodominant peptide (**S1 Fig**). None of the peptides showed a clear discrimination between the *Ascaris Lumbricoides* infected and uninfected group (**Table 3**).

**Table 3:** Diagnostic performance of selected peptides.

Pathogen	Peptide name	Peptide	IgM Median		IgG Median	
			AUC	p-value	AUC	p-value
*Ascaris lumbricoides*	ACJ03764.1-3852-38-52	KQIITGAPDKTDAEI	0.4428224	0.847	0.3746959	0.667
	AAD13652.1-133-147	RRHHFTLESSLDTHL	0.5136032	0.655	0.540146	0.173
	AAD13652.1-142-156	SLDTHLKWLSQEQKD	0.4190998	0.571	0.3527981	0.712
	ACJ03763.1-50-64	TDPEIEADIDAFVAK	0.5346715	0.270	0.5851582	0.061
	CAA60047.1-553-568	TMKIYARDQGGIHNPP	0.4890511	0.438	0.080292	0.361
*Necator americanus*	AAP41952.1-180-192	AGNMMGKDIYEKG	0.2278912	0.34	0.7278912	0.218
	CAC00543.1-123-135	SRKCPFYDIACML	0.00000	0.826	0.00000	0.686
	AAP41952.1-194-206	PCSKCENCDKEKG	0.4115646	0.335	0	0.653
*S*. *haematobium*	XP_035588858.1-206-220	EDSDEDDSTVYEVAM	0.5110029	0.811	0.5777417	0.037
	XP_035587815.1-269-283	EISLDPIYKPEDLCI	0.485318	0.765	0.5435057	0.135
	XP_012797374.1-78-92	NHIKTVQSGREPDLP	0.3930375	0.572	0.3919553	0.340
	AAZ29530.1-25-29	PINHGPKDVSIQTYP	0.5373107	0.219	0.4440795	0.58
	XP_012799745.1-16-30	SFLEMDADNNEMIDK	0.4098124	0.306	0.4451659	0.954
*S*. *mansoni*	P20287.1-58-72	GEVSTENGKLKVNGK	0.2385246	0.215	0.1920765	0.001
	AAA29903.1-222-237	KSDNQIKAVPASQAL	0.1948087	0.124	0.1295082	0.062
	P09841.3-6-20	LFLISILHILLVKCQ	0.2928962	0.739	0.097541	0.389
	AAA29900.1-145-159	CGAKGPDDYRGNVPA	0.3770489	0.215	0.423224	0.001
	P09792.1-29-43	VDYEDERISFQDWPK	0.3612022	0.020	0.3800546	0.052
*Trichuris trichiura*	CDW57769.1-659-673	DMLIKARTNVFAVNK	0.3888889	0.007	0.3569444	0.236
	CDW57769.1-2017-2031	RPEYKDKECYLEHDE	0.5347222	0.792	0.3722222	0.343
	CDW57769.1-834-848	STLDQWRDHLEKLFA	0.5875	0.304	0.1722222	0.069
	CDW52482.1-326-340	TNEVWEAWTILDDYI	0.4601399	0.744	0.5482517	0.710
	CDW57769.1-1518-1532	VRYESFRVAADDFLD	0.4569444	0.744	0.525	0.833

### Antibody reactivity and discrimination of *Necator americanus* infection

Peptide AAP41952.1-180-192 (AGNMMGKDIYEKG) was the only reactive peptide for IgM reacting with 6 samples from the negative control group with highest response being 2112. 5 RFU. For IgG no reactivity was observed with fluorescence intensity less than 400 RFU for all the *Necator americanus* derived peptide in the *Necator americanus* infected and uninfected groups (**S1 Fig**).

Like the heat maps for *Ascaris lumbricoides* derived peptides there was no immunodominant peptide for the *Necator americanus* peptides and none of the peptides showed a clear discrimination between the *Necator americanus* infected and uninfected groups (**Table 3**).

### Antibody reactivity and discrimination of *S*. *haematobium* infection

*S*. *haematobium* derived peptides were all reactive with IgG with high fluorescence intensities observed in the infected group compared to the uninfected group across all the peptides. Peptide XP_012799745.1-16-30 (SFLEMDADNNEEMIDK) gave the highest response 8576 RFU and by observing the heat maps XP_035588858.1-206-220 (EDSDEDDSTVYEVAM) appeared to be the immunodominant peptide for the *S*. *haematobium* derived peptides. Peptide XP_035588858.1-206-220 showed discrimination between the *S*. *haematobium* infected and uninfected group p<0.037, however it had an AUC of 0.5777417 (**S1 Fig**). Likewise, all *S*. *haematobium* derived peptides reacted with IgM with high fluorescence intensities observed in the infected group compared to the uninfected group across all peptides. Peptide XP_035588858.1-206-220 (EDSDEDDSTVYEVAM) was observed to be the immunodominant peptide and it gave the highest response of 12610 RFU. However, none of the peptides showed a clear discrimination between infected and uninfected groups including XP_035588858.1-206-220 (**Table 3**).

### Antibody reactivity and discrimination of *S*. *mansoni* infection

Peptide microarray technology exhibited levels of IgM reactivity against peptides derived from *S*. *mansoni* antigens for both *S*. *mansoni* infected and uninfected groups, with the exception of AAA29903.1-222-237 which did not react with the *S*. *mansoni* uninfected group. For IgG the technology exhibited reactivity for three peptides; P09792.1-29-43 (VDYEDERISFQDWPK) (reacting with 3 samples from the uninfected group), P20287.1-58-72 (GEVSTENGKLKVNGK) (reacting with 1 sample from the uninfected group) and AAA29903.1-222-237 (KSDNQIKAVPASQAL) (reacting with 1 sample from the infected group) with RFU values of 1482.25, 1144 and 532.5, respectively. Examination of the heat maps revealed that peptide P20287.1-58-72 (GEVSTENGKLKVNGK) was the immunodominant peptide for both IgG and IgM (**S1 Fig**). However, none of the peptides showed a clear discrimination between *S*. *mansoni* infected and the uninfected groups **(Table 3**).

### Antibody reactivity and discrimination of *Trichuris trichiura* infection

Peptide CDW57769.1-659-673 (DNLIKARTNVFAVNK) was the only *Trichuris trichiura* derived peptide that was not reactive with IgG and peptide CDW52482.1-326-340 (TNEVWEAWTILDDYI) gave the highest RFU value of 8572 with sera from the *Trichuris trichiura* uninfected group. For IgM, all the peptides showed immunoreactivity with fluorescence intensities above than 500 RFU for all the peptide in the *Trichuris trichiura* infected and uninfected groups. Visual inspection of the heat maps showed that peptide CDW52482.1-326-340 was immunodominant for IgG and peptide CDW57769.1-2017-2031 (RPEYKDKECYLEHDE) and peptide CDW57769.1-1518-1532 (VRYESFRVAADDFLD) were immunodominant for IgM (**S1 Fig**). None of the peptides showed a clear discrimination between the *Trichuris trichiura* infected and uninfected group (**Table 3**).

### Antibody reactivity against peptides derived from *Bacillus anthracis* proteins

Peptide microarray technology showed IgG reactivity against two peptides derived from *Bacillus anthracis* antigens, WP_001022096.1-35-44 (GGHGDVGMHV) and WP_040119992.1-736-358 (IINPSENGDTSTNGIKK) with RFU values of 1034 and 665.25, respectively. For IgM all the peptides were responsive and WP_001022096.1-35-44 gave the highest fluorescence intensity value of 2502.5 RFU. The heat maps indicated that peptide WP_001022096.1-35-44 appeared to be immunodominant for pathogens (**S2 Fig**).

### Antibody reactivity against peptides derived from *Mycobacterium leprae* proteins

Peptide microarray technology showed that all *Mycobacterium leprae* derived peptides were responsive with at least one plasma or serum sample for both IgG and IgM. Peptide WP_010907696.1-11-25 (DKILVQAGEAETMTP) gave the highest fluorescence intensity for both IgG (2869 RFU) and IgM (7803.5 RFU). The heat maps indicated that peptide CAA37572.1-10-24 (EIDLTNKNAAKLRGD) was immunodominant for both IgG and IgM (**S2 Fig**).

### Antibody reactivity against peptides derived from *Wuchereria bancrofti* proteins

Peptide microarray technology showed that both IgG and IgM were reactive with *Wuchereria bancrofti* derived peptides except for AAC35355.1-55-68 (EVVETDGKKKECSS) which had a fluorescent intensity of 494 RFU for IgG. Peptide AAC35355.1-46-60 (GGDEYVTKGEVVETD) gave the highest fluorescence intensity for both IgG (6680.75 RFU) and IgM (3111 RFU). The heat maps indicate that peptide AAC35355.1-46-60 was also immunodominant for both IgG and IgM (**S2 Fig**).

### Antibody reactivity against peptides derived from *Rabies lyssavirus* proteins

Peptide microarray technology showed that only one *Rabies lyssavirus* peptide BAJ04981.1-12-26 (QYEYKYPAIKDLKKP) was reactive with IgG with a fluorescence intensity of 1048 RFU. For IgM all peptides were reactive and QEJ74712.1-99-113 (NVGVQIVRQMRSGER) gave the highest fluorescence intensity (1872 RFU). The heat maps indicate that peptide QEJ74712.1-99-113 was also immunodominant for IgM (**S2 Fig**).

### Antibody reactivity against peptides derived from *Chlamydia trachomatis* proteins

Peptide microarray technology exhibited levels of IgM reactivity against all peptides derived from *Chlymadia trachomatis*. For IgG the technology exhibited reactivity against three peptides; P19542.1-261-270 (TKDASIDYHE), AAA23156.1-73-81 (VLKTDVNKE) and AAA23156.1-381-390 (TRLIDERAAH) with RFU values of 598.25, 1324.5 and 908.75 respectively. Peptide P19542.1-261-270 exhibited the highest response for IgM with fluorescence intensity of 5808 RFU. Peptide P19542.1-261-270 was also immunodominant for both IgG and IgM compared to the other peptides (**S2 Fig**).

### Antibody reactivity against peptides derived from *Trypanosoma brucei* proteins

Peptide microarray technology showed that only one *Trypanosoma brucei* derived peptide XP_011774209.1-132-145 (SAIHATKTVDIKAL) was reactive with IgG with a fluorescence intensity of 1629.5 RFU. For IgM the technology exhibited reactivity against three peptides; XP_844815.1-369-382 (SSKIKESKVILMAV) (reacting with one serum sample), APD74596.1-96-110 (ENREKWEADKKLIVA) (reacting with 5 serum samples) and CAC33895.1-78-92 (ETDFKATADDNKKPQ) with RFU values of 2069.5, 1266.25 and 822.25 respectively. Peptide XP_844815.1-369-382 exhibited the highest response for IgM with fluorescence intensity of 2069.5 RFU. Peptide XP_844815.1-369-382 was also immunodominant for both IgG and IgM compared to the other peptides (**S2 Fig**).

## Discussion

In a time of fierce competition for global health funding, surveillance of a single infection is difficult to defend as a priority [19]. Accurate surveillance of multiple-infections enable public health officials to identify opportunities for implementation of integrated control programs [20]. NTDs multiplex peptide microarray tools could represent an integrated surveillance platform that could be used for monitoring exposure and for disease diagnosis. With this background, B-cell linear epitopes derived from the antigenic proteins of *Ascaris lumbricoides*, *Necator americanus*, *S*. *haematobium*, *S*. *mansoni*, *Trichuris trichiura*, *Bacillus anthracis*, *Mycobacterium leprae*, *Wuchereria Bancrofti*, *Rabies lyssavirus*, *Chlamydia trachomatis* and *Trypanosoma brucei* were selected for peptide microarray technology. Plasma and serum samples from NTDs endemic areas were evaluated on the peptide microarray to detect IgG and IgM antibodies as described in the experimental section. Species sero-reactivity observed with both IgG and IgM was indicative of exposure to the different NTDs parasites antigens in Murewa, Makoni and Shamva rural districts.

As a typical finding of peptide microarray immunoassays, the signal distribution was highly skewed as was described by Hecker and colleagues [21]; many peptides were detected with high signals for several samples, with no obvious difference between the infected and uninfected groups. The results were in agreement with Odegaard and Hsieh, 2014 who demonstrated that exposure is universal in schistosomiasis endemic areas as defined by the ubiquity of schistosomiasis specific antibodies [22].

Comparative studies between IgM and IgG antibody detection in endemic areas have showed significant differences in their diagnostic capabilities, demonstrating a higher IgM detection in a low endemic setting, without extensive knowledge of the particular infective conditions in studied individuals [23]. In the present study AUC was chosen to summarize the overall diagnostic accuracy of IgG and IgM for *Ascaris lumbricoides*, *Necator americanus*, *S*. *haematobium*, *S*. *mansoni* and *Trichuris trichiura* derived peptides. AUC was chosen because it is a classification-threshold-invariant that measures the quality of model predictions irrespective of classification threshold. AUC takes values from 0 to 1, where a value of 0 indicates a perfectly inaccurate test and a value of 1 reflects a perfectly accurate test. According to AUC values most of the peptides selected were inaccurate (that is they falsely diagnosed villagers infected or uninfected with disease) with AUC values less than 0.5. Two peptides CAC00543.1-123-135 and AAP41952.1-194-206 were perfectly inaccurate (100% wrong) in predicting villagers with disease and those without disease. Some peptides had AUC values ranging from 0.5 to 0.5875 for both IgM and IgG suggesting no discrimination (that is no ability to diagnose villagers with and without the disease or condition based on the peptides) [24, 25].

Justifications for the lack of discrimination between the infected and uninfected groups by the peptides selected in the study are multi-fold. Just like most antibody detection assays peptide microarray immunoassays does not allow for differentiation of acute or previous infections nor discrimination between persisting antibodies and reinfection. Several studies have demonstrated that antibody detection test may not differentiate between active, prior infection or re-infection because antibodies may persist for many months to years after successful treatment in most of the NTDs [26, 27]. Hinz and colleagues (2017) suggested that remaining or increasing antibody level limits the usefulness of serological tests. In some individuals negative or intermediate results may be misleading due to a low level of antibody response and late or absent seroconversion or an age-dependent decreasing antibody response in people from endemic regions, resulting from cumulative exposure to schistosomes [27].

Commonly used diagnostic methods for schistosomiasis and STH have low sensitivity for the detection of light infections; and many light infections are missed due to absence of eggs in urine and stool specimens [28–31]. Compared to parasitological diagnosis, serology provides more sensitive tools for the diagnosis of helminths, especially in infections with low intensity [27]. The above-mentioned points may have resulted in the failure of the peptides in diagnosing schistosomiasis and STHs.

### Limitations and recommendations

Sero-reactivity of at least one peptide derived from each NTD with at least one sample was indicative of the presence of these NTDs in the study population, however, we would need to include sera from uninfected individual from non-endemic areas with no history of exposure to determine the seroprevalence of the NTDs.

A clear limitation of conventional peptide microarrays is their restriction to linear protein epitopes, whereas conformational epitope antibody recognition cannot be identified [32]. Detection of antibodies recognizing all potential epitopes whether linear, conformational or carbohydrate or LPS is a key requirement to comprehensively profile the humoral immune response [33]. Studies have shown that sera of individuals infected with helminths such as *S*. *haematobium* and *S*. *mansoni* contain IgG and IgM antibodies against defined carbohydrate epitopes [34, 35]. This meant that the peptide microarray technology could not detect antibodies that bind non-protein epitopes. In future peptide microarray studies, we recommend including both linear and conformational protein epitope peptide for evaluation.

The peptide microarray technology described in this study is too complex and expensive for routine clinical microbiology. We recommend that the peptides established by the technology be transferred to a wide range of platforms including, enzyme-linked immunosorbent assay, radioimmunoassay, lateral flow, western blot, and bead-based assays, where they may facilitate diagnostics, epidemiology, and vaccinology. Lateral flow assay can be used in field settings in low resource countries.

## Conclusion

Although the selected peptides had poor diagnostic performances, species-specific sero-reactivity was indicative of exposure to the different parasitic antigens in the study population. Peptide microarray immunoassay demonstrated that more individuals in Shamva, Murewa and Makoni rural districts in Zimbabwe were exposed to schistosomiasis and STHs than the expected infection prevalence. Peptide microarrays maybe valuable tools for integrated NTDs surveillance and for screening parasites exposure in endemic areas.

## Supporting information

S1 Fig Heat maps and bar graphs generated from peptide microarray data for *A*. *lumbricoides*, *N*. *americanus*, *S*. *haematobium*, *S*. *mansoni* and *T*. *trichiura*. Samples are arranged in rows and infection status shown on the left key. Peptides shown by their protein accession number and sequence position are arranged in columns. Bar graphs representing the peptide reactivity for each serum and plasma in both the infected and uninfected groups in the study.
(PDF)

S2 Fig Heat maps and bar graphs generated from peptide microarray data for *B*. *anthracis*, *M*. *Leprae*, *W*. *bancrofti*, *Rabies lyssavirus*, *C*. *trachomatis* and *T*. *brucei*. Samples are arranged in rows and infection status shown on the left key. Peptides shown by their protein accession number and sequence position are arranged in columns. Bar graphs representing the peptide reactivity for each serum and plasma in both the infected and uninfected groups in the study.
(PDF)

S1 Table Area under the ROC curve and the diagnostic performance of each peptide. (PDF)

S2 Table Antibody reactivity against the peptide microarray internal quality control spots. (XLSX)

S1 File Peptide microarray immunoassay data set. (RAR)
