# Supplementary material for: Multiplex peptide microarray profiling of antibody reactivity against neglected tropical diseases derived B-cell epitopes for serodiagnosis in Zimbabwe
Source: PLoS One. 2022 Jul 22;17(7):e0271916. doi: 10.1371/journal.pone.0271916 (PMC9307155; doi:10.1371/journal.pone.0271916)
Supplement: S1 Fig — Samples are arranged in rows and infection status shown on the left key. Peptides shown by their protein accession number and sequence position are arranged in columns. Bar graphs representing the peptide reactivity for each serum and plasma in both the infected and uninfected groups in the study. (PDF) [file pone.0271916.s001.pdf]

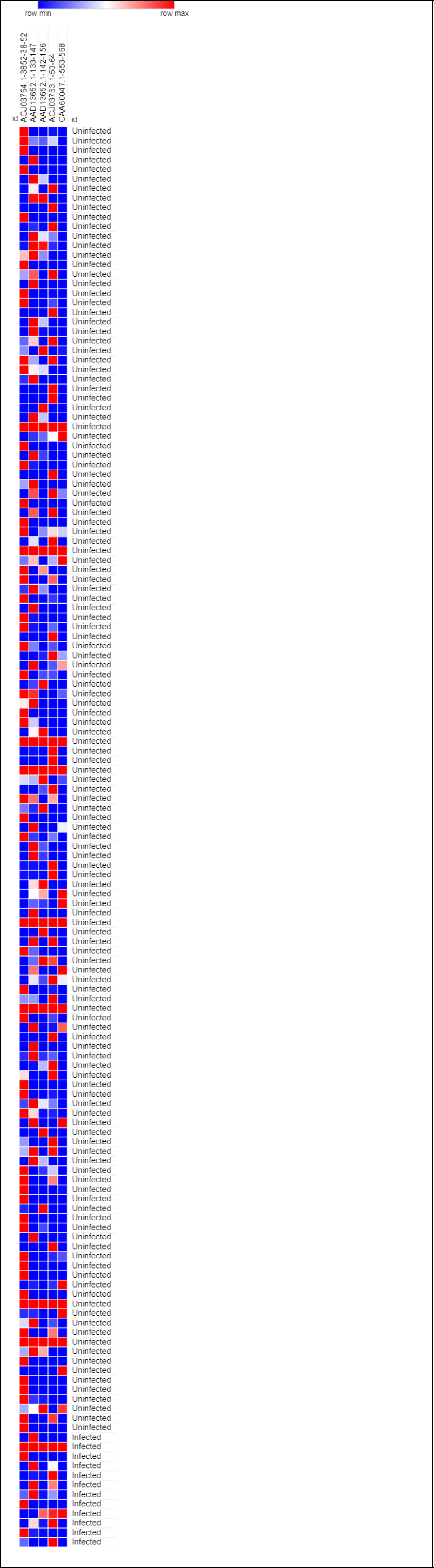

Ascaris lumbricoides IgG heat map

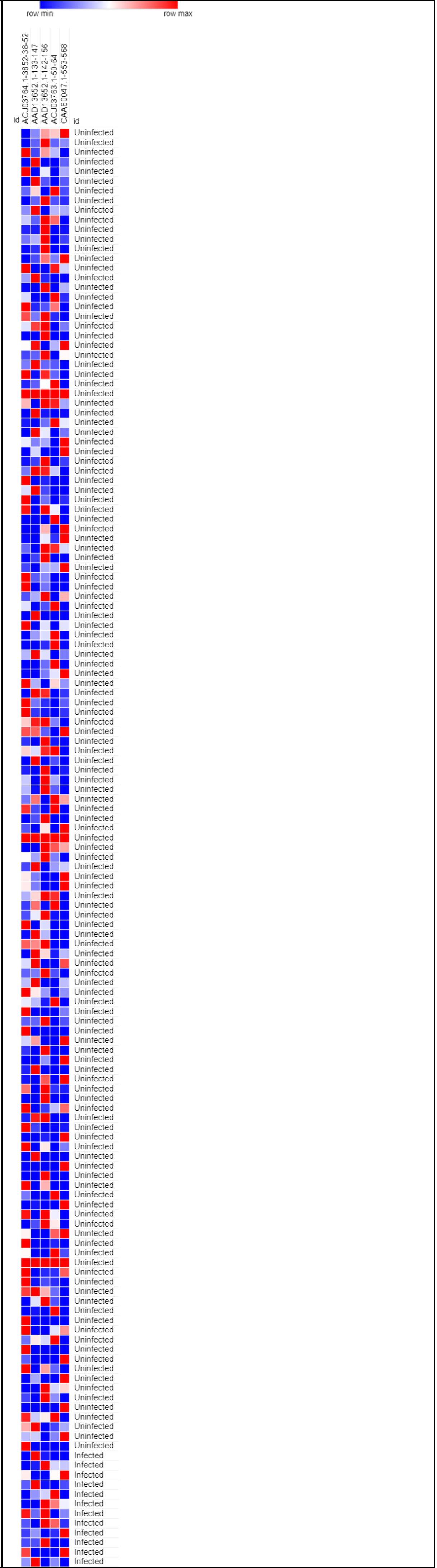

Ascaris lumbricoides IgM heat map

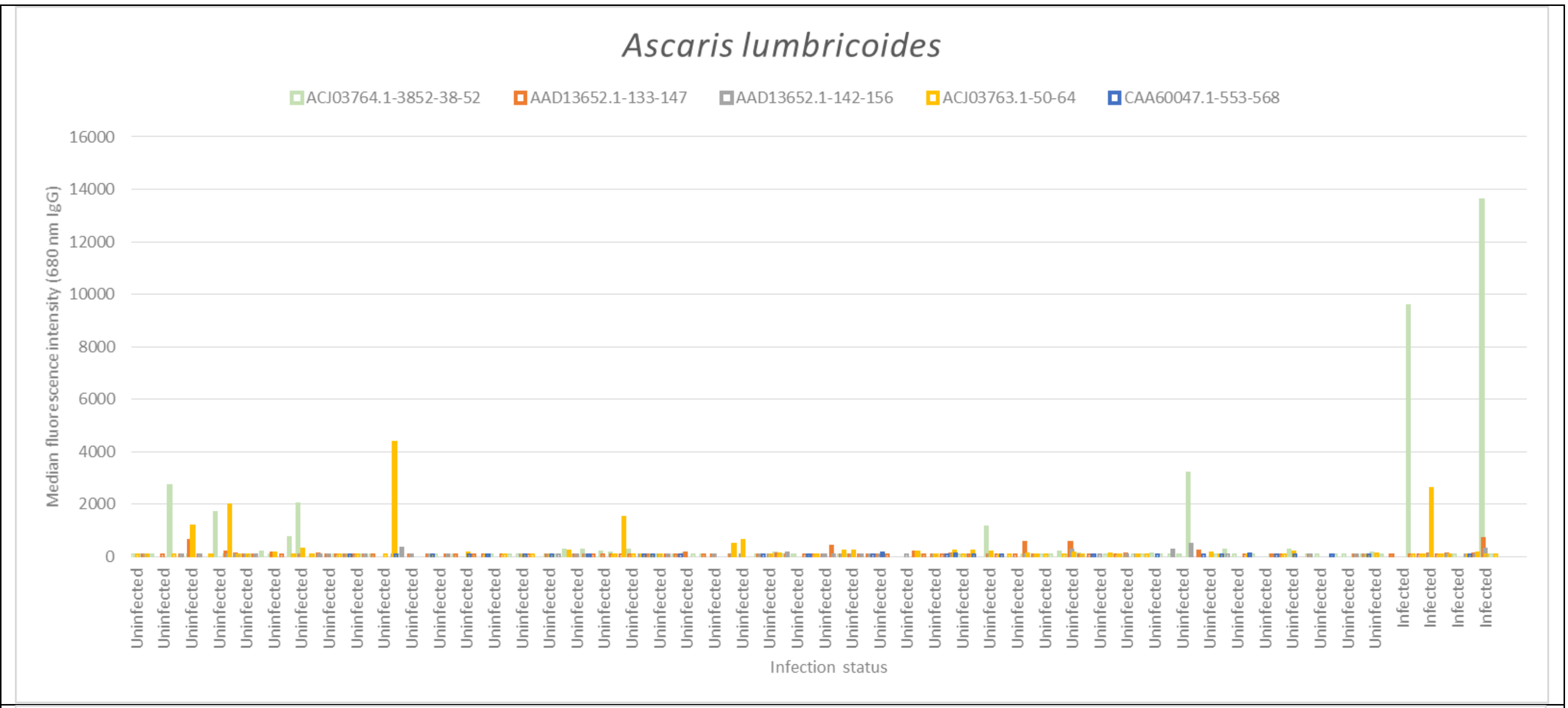

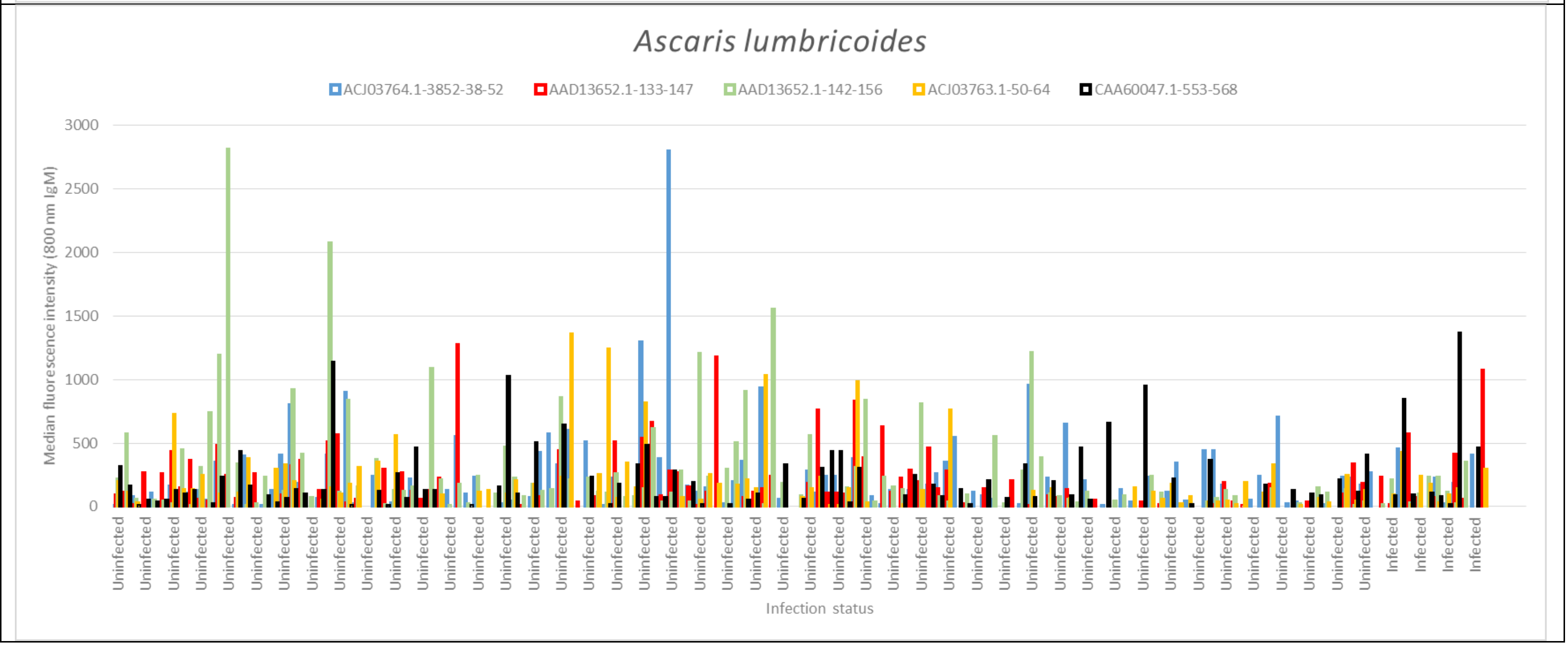

: Heat maps and bar graphs generated from peptide microarray data for *Ascaris lumbricoides* A. Heat maps generated from peptide microarray. Samples are arranged in rows and infection status shown on the left key. Peptides shown by their protein accession number and sequence position are arranged in columns. B. Bar graphs representing the peptide reactivity for each serum and plasma in both the infected and uninfected groups in the study.

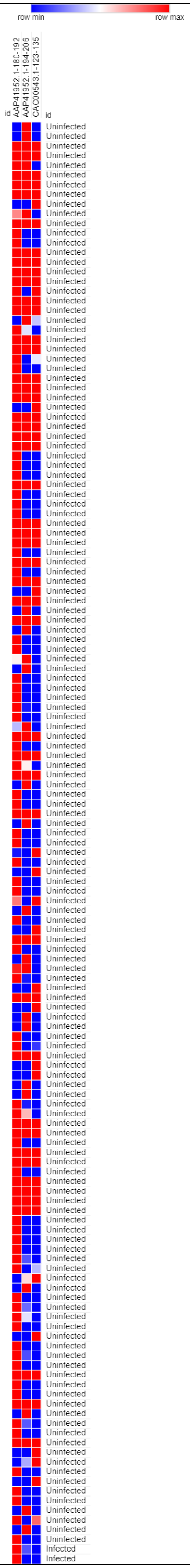

Necator americanus IgG heat map

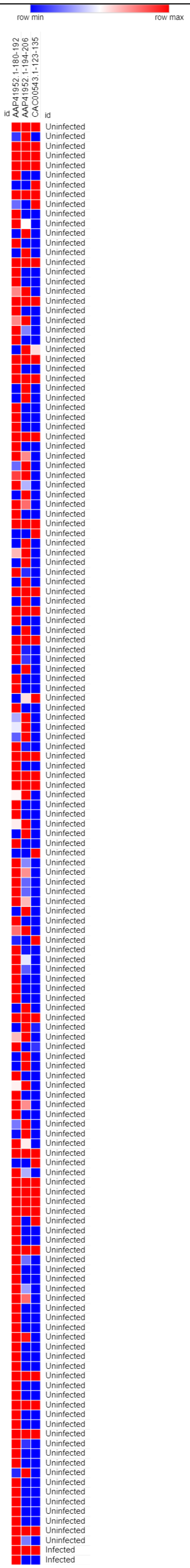

Necator americanus IgM heat map

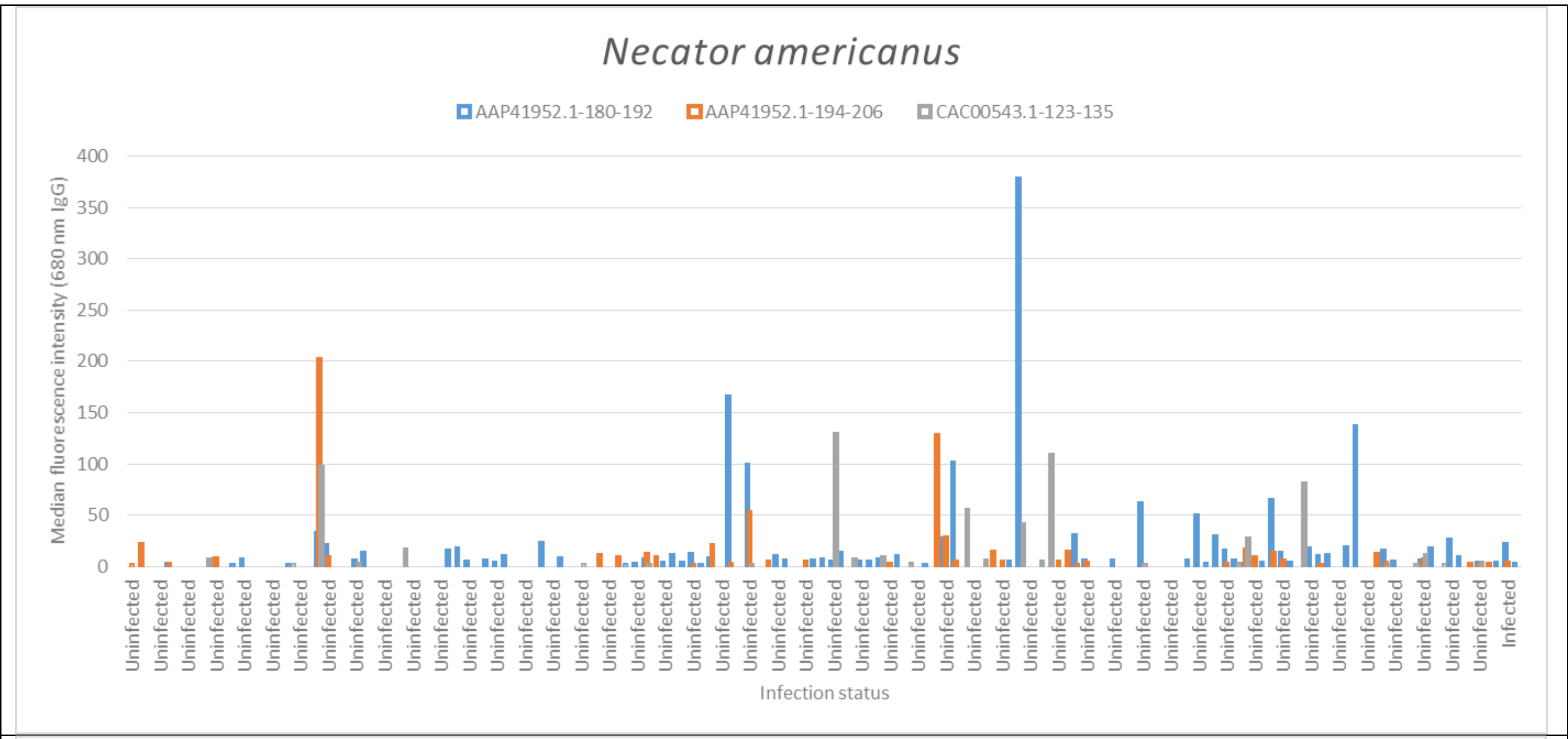

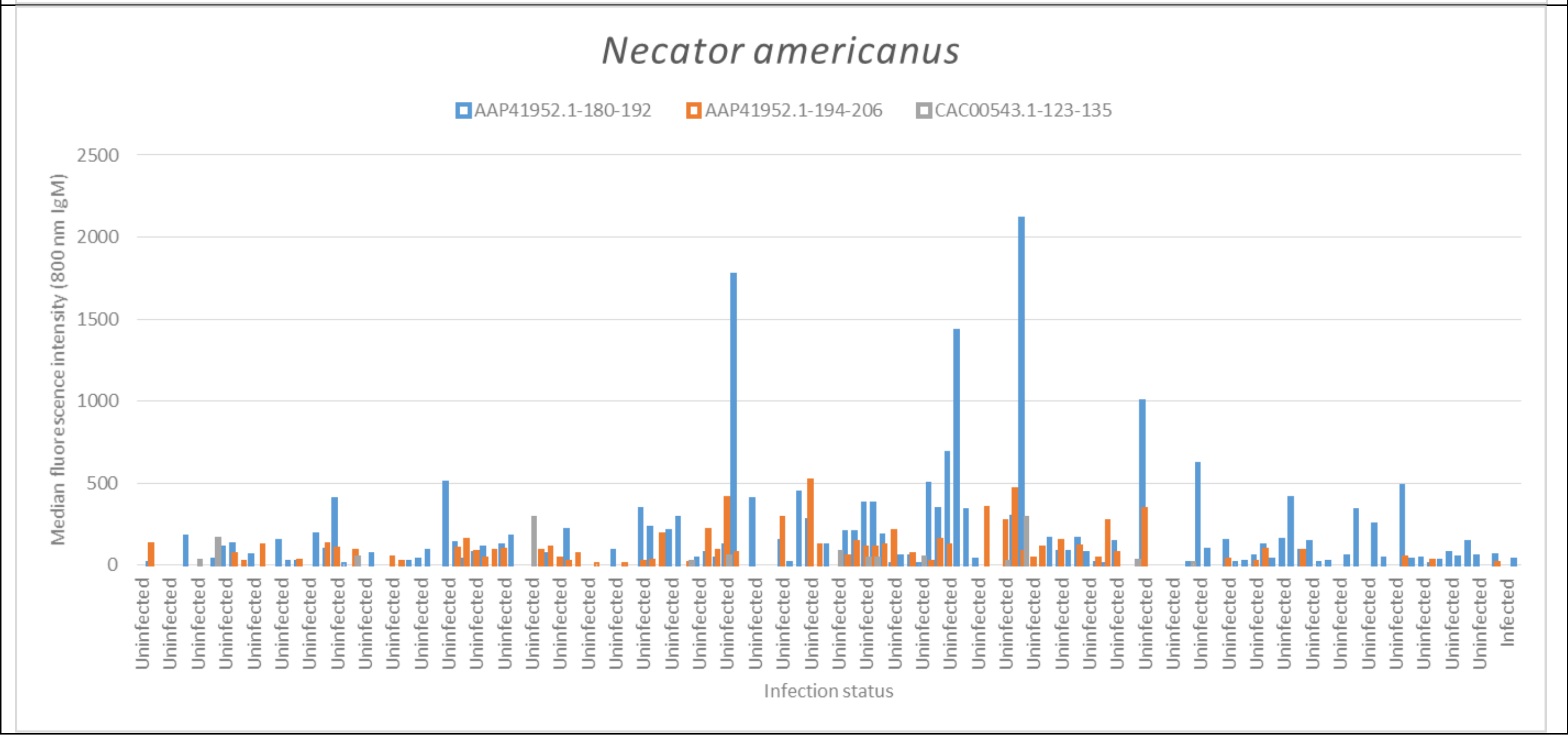

: Heat maps and bar graphs generated from peptide microarray data for *Necator americanus* A. Heat maps generated from peptide microarray. Samples are arranged in rows and infection status shown on the left key. Peptides shown by their protein accession number and sequence position are arranged in columns. B. Bar graphs representing the peptide reactivity for each serum and plasma in both the infected and uninfected groups in the study.

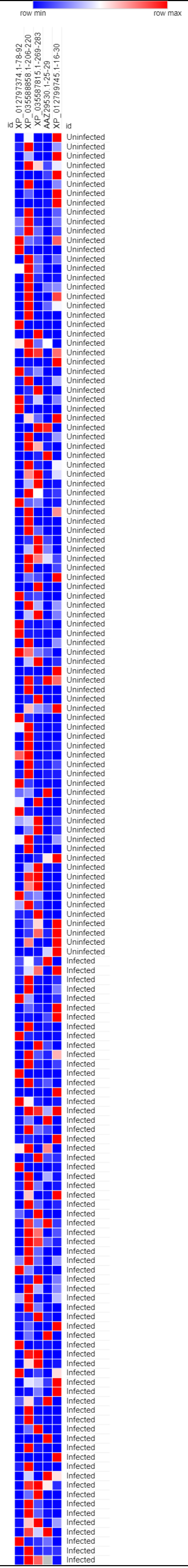

S. haematobium IgG heat map

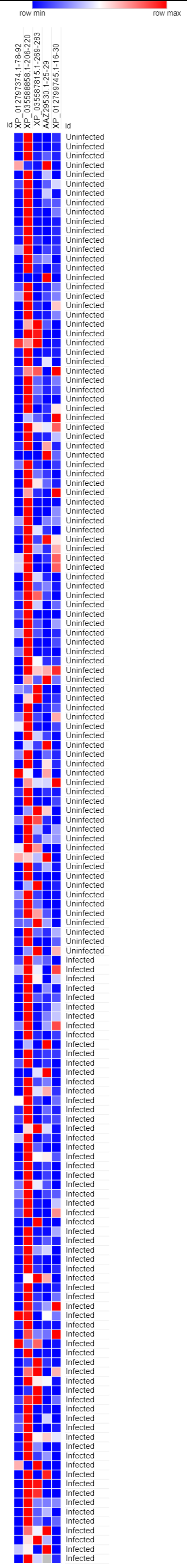

S. haematobium IgM heat map

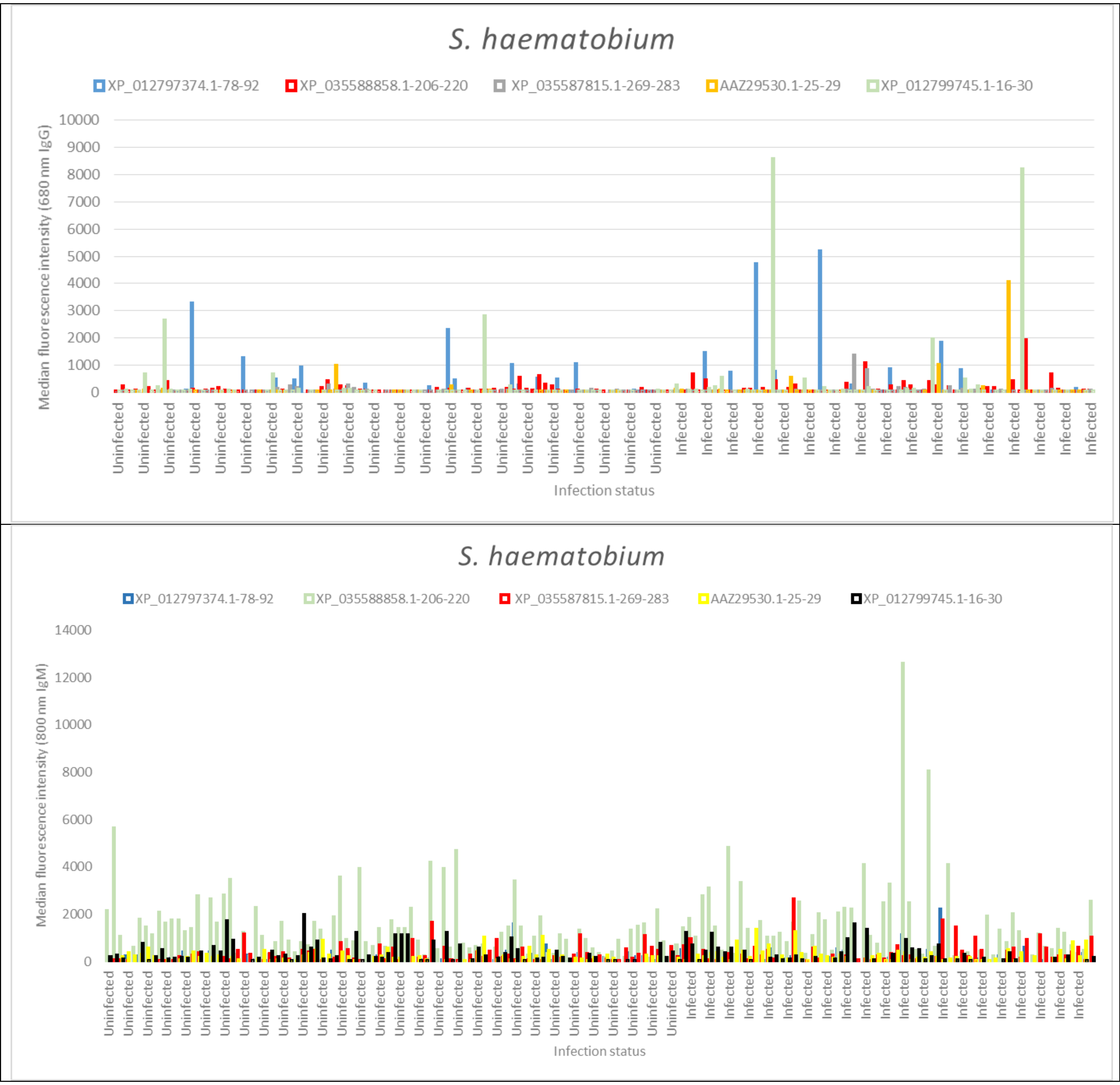

: Heat maps and bar graphs generated from peptide microarray data for *S. haematobium* A. Heat maps generated from peptide microarray. Samples are arranged in rows and infection status shown on the left key. Peptides shown by their protein accession number and sequence position are arranged in columns. B. Bar graphs representing the peptide reactivity for each serum and plasma in both the infected and uninfected groups in the study.

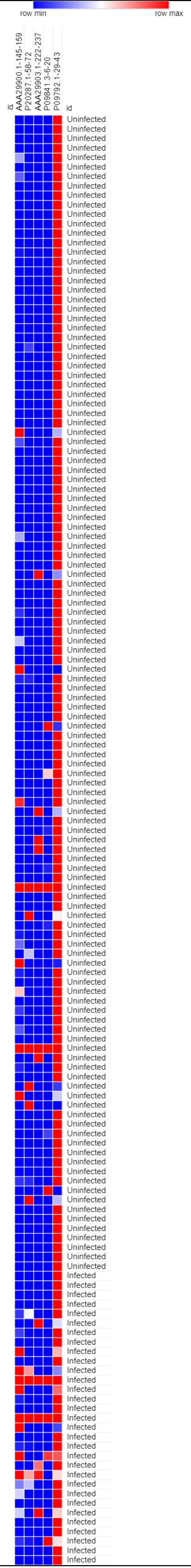

S. mansoni IgG heat map

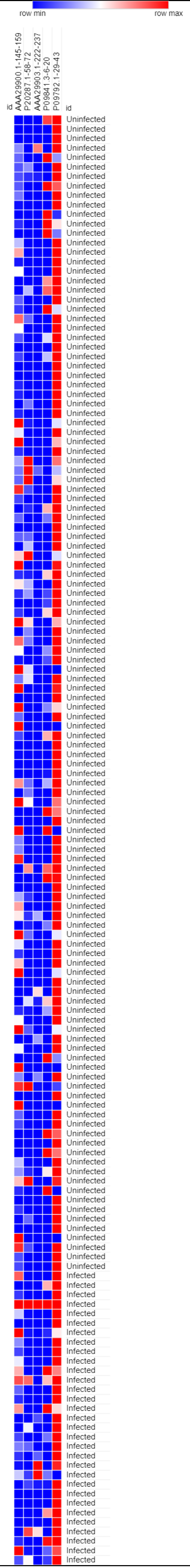

S. mansoni IgM heat map

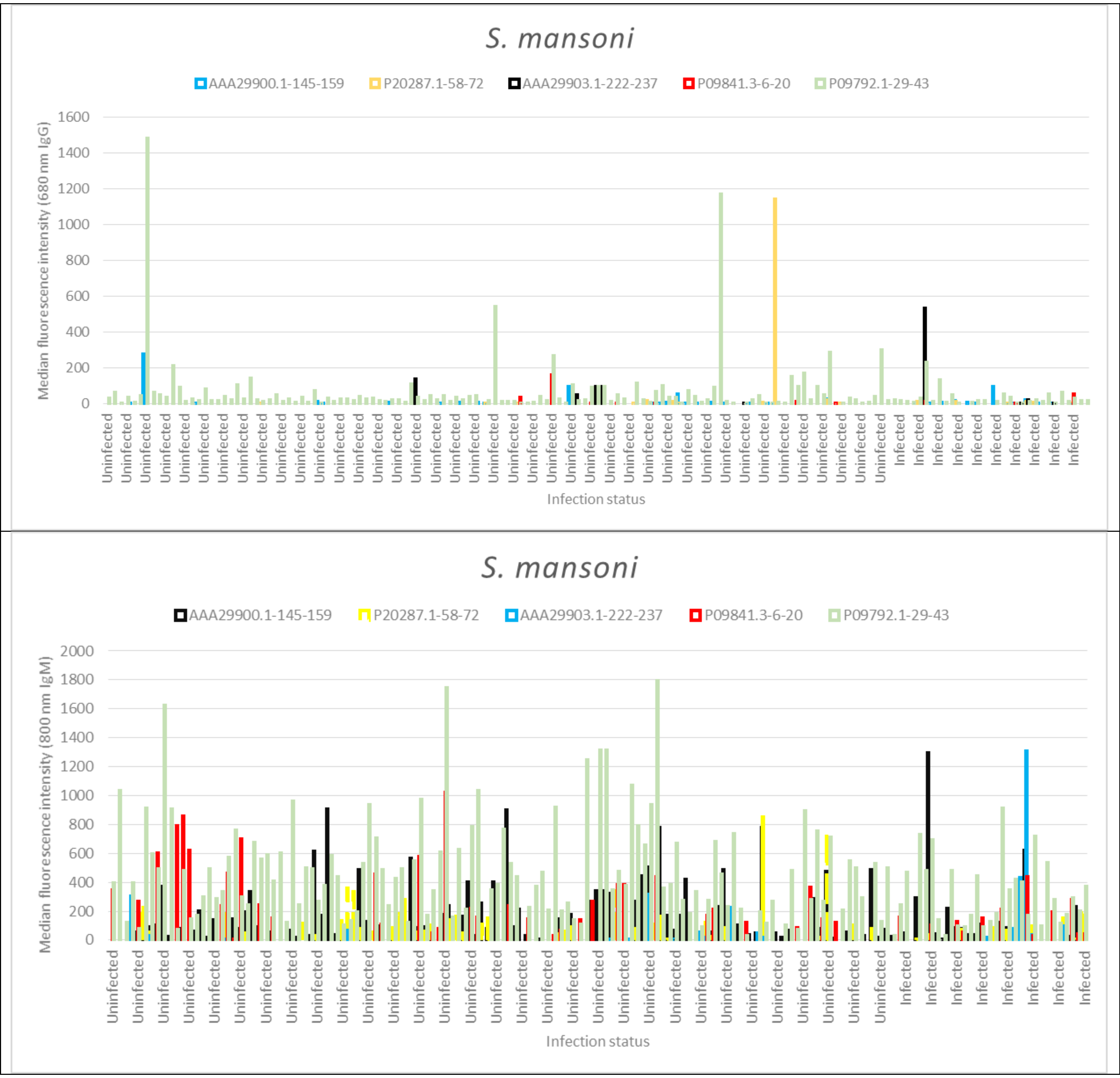

: Heat maps and bar graphs generated from peptide microarray data for *S. mansoni* A. Heat maps generated from peptide microarray. Samples are arranged in rows and infection status shown on the left key. Peptides shown by their protein accession number and sequence position are arranged in columns. B. Bar graphs representing the peptide reactivity for each serum and plasma in both the infected and uninfected groups in the study.

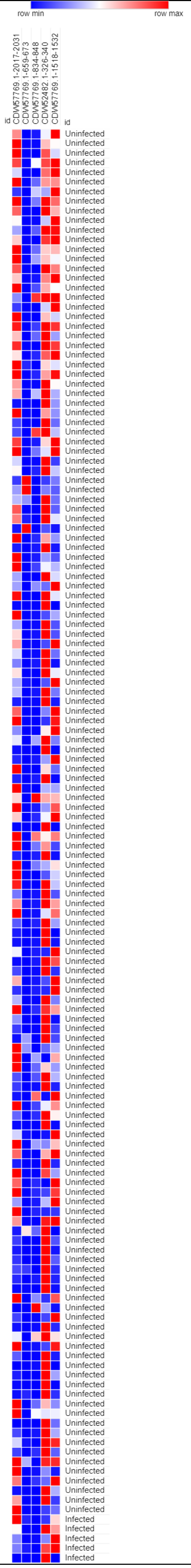

Trichuris trichuria IgG heat map

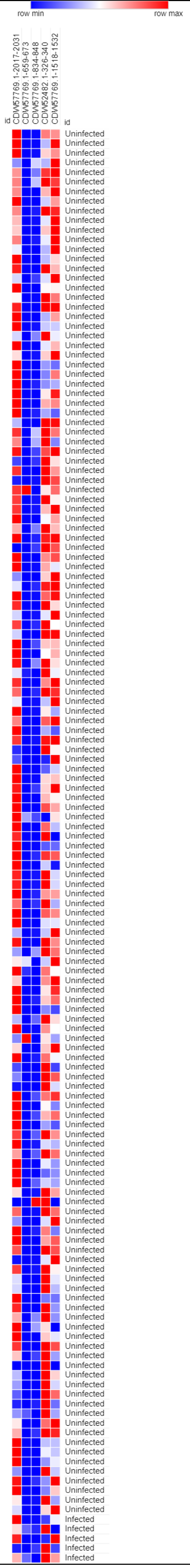

Trichuris trichuria IgM heat map

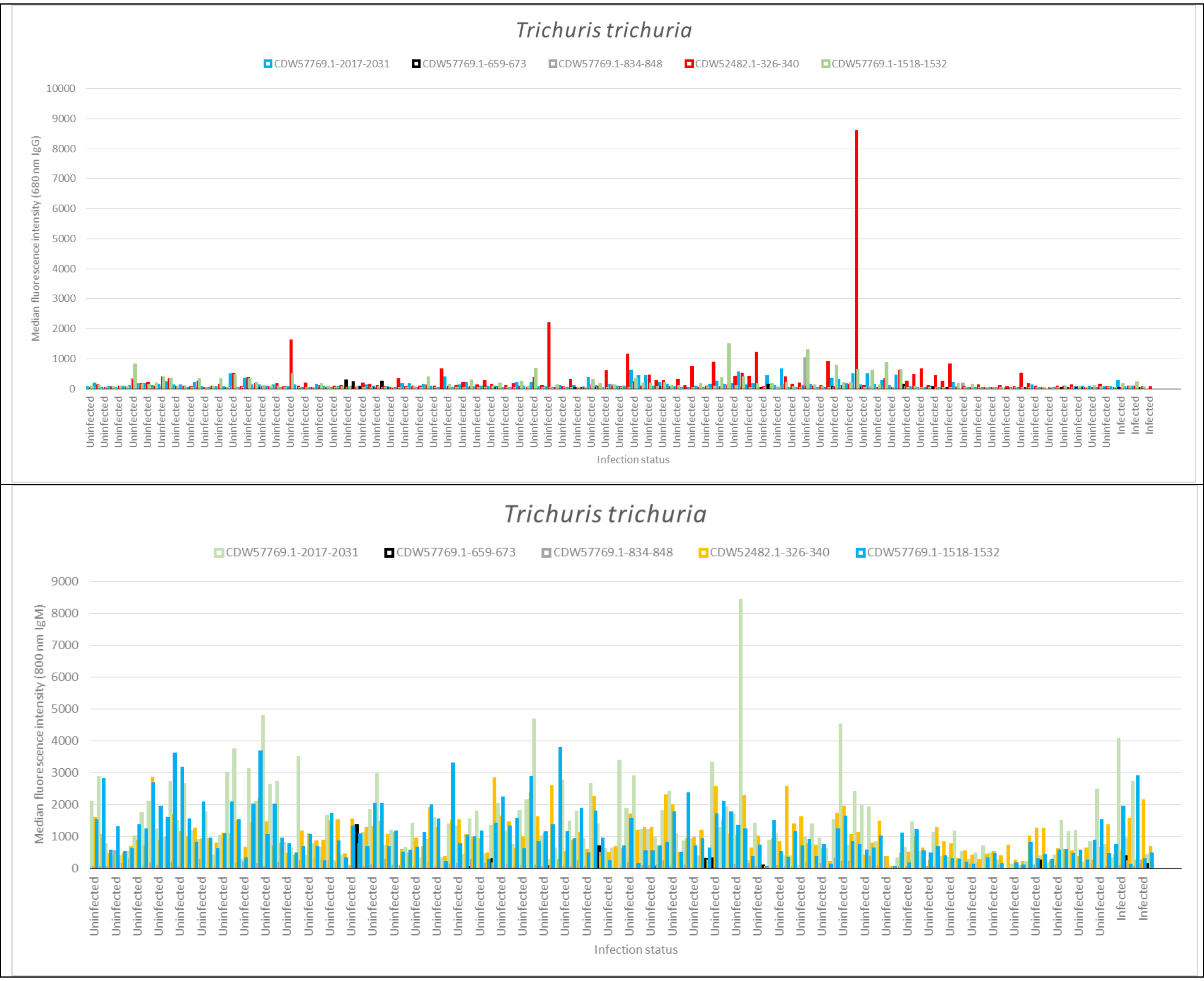

: Heat maps and bar graphs generated from peptide microarray data for *Trichuris trichuria* A. Heat maps generated from peptide microarray. Samples are arranged in rows and infection status shown on the left key. Peptides shown by their protein accession number and sequence position are arranged in columns. B. Bar graphs representing the peptide reactivity for each serum and plasma in both the infected and uninfected groups in the study.
