# Supplementary material for: Multiplex peptide microarray profiling of antibody reactivity against neglected tropical diseases derived B-cell epitopes for serodiagnosis in Zimbabwe
Source: PLoS One. 2022 Jul 22;17(7):e0271916. doi: 10.1371/journal.pone.0271916 (PMC9307155; doi:10.1371/journal.pone.0271916)
Supplement: S2 Fig — Samples are arranged in rows and infection status shown on the left key. Peptides shown by their protein accession number and sequence position are arranged in columns. Bar graphs representing the peptide reactivity for each serum and plasma in both the infected and uninfected groups in the study. (PDF) [file pone.0271916.s002.pdf]

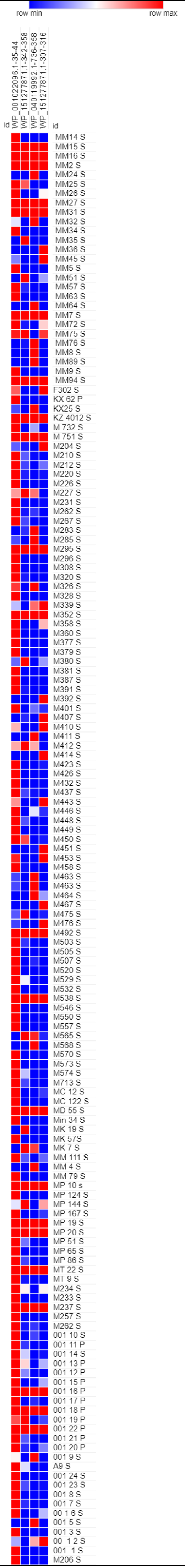

Bacillus anthracis IgG heat map

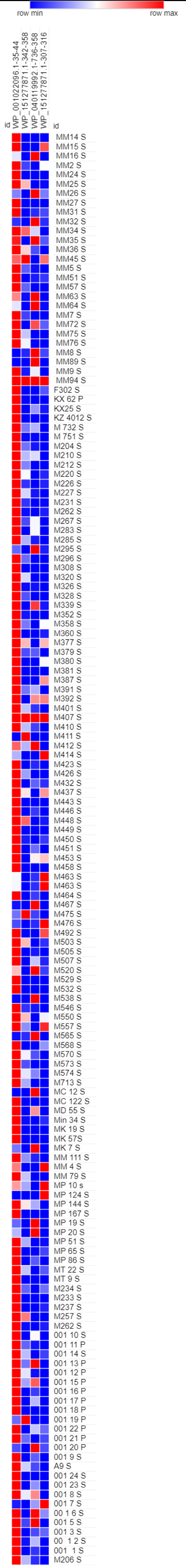

Bacillus anthracis IgM heat map

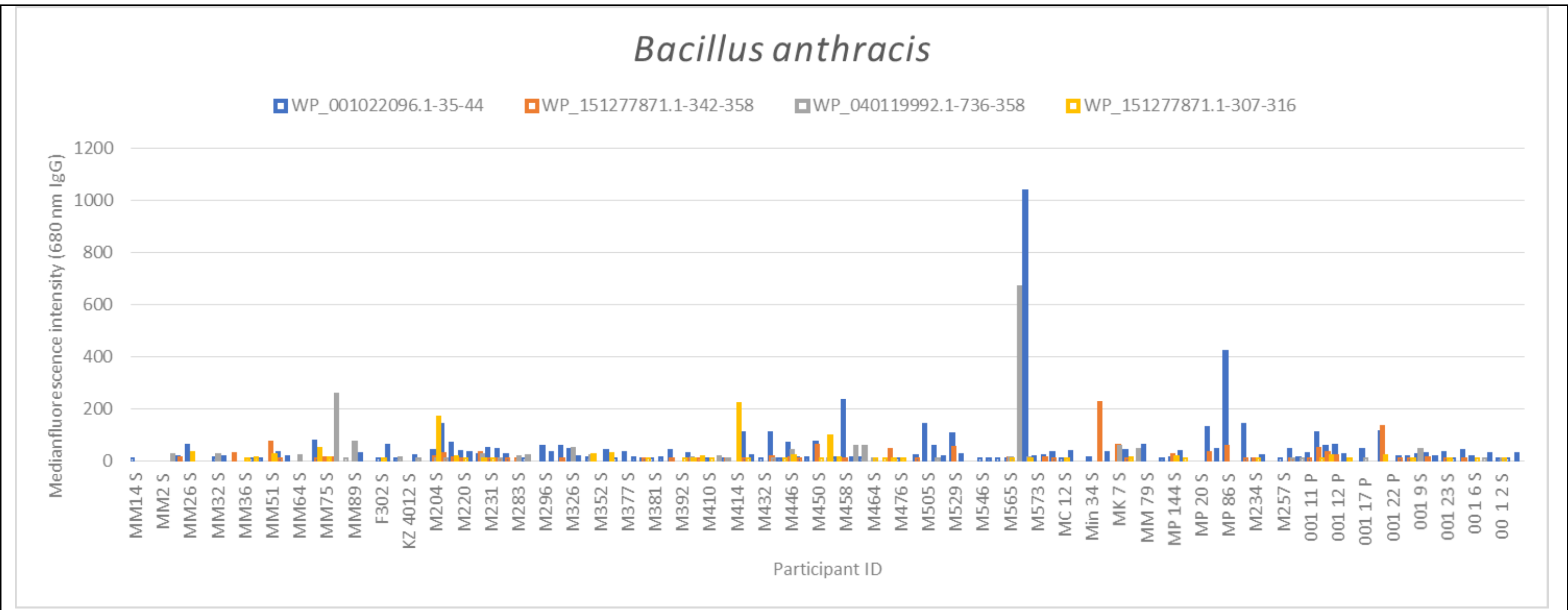

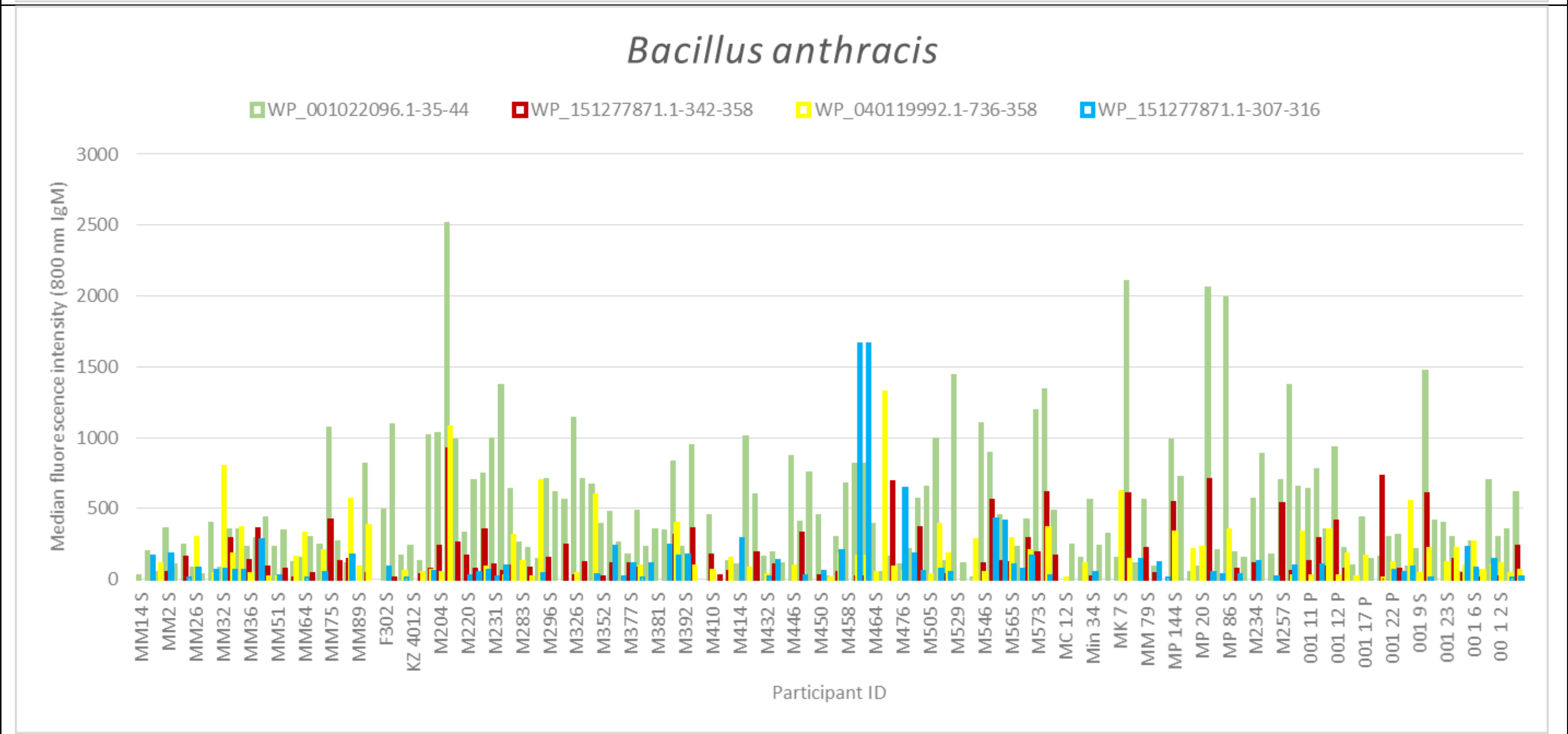

Heat maps and bar graphs generated from peptide microarray data for *Bacillus anthracis* A. Heat maps generated from peptide microarray. Samples are arranged in rows and infection status shown on the left key. Peptides shown by their protein accession number and sequence position are arranged in columns. B. Bar graphs representing the peptide reactivity for each serum and plasma in both the infected and uninfected groups in the study.

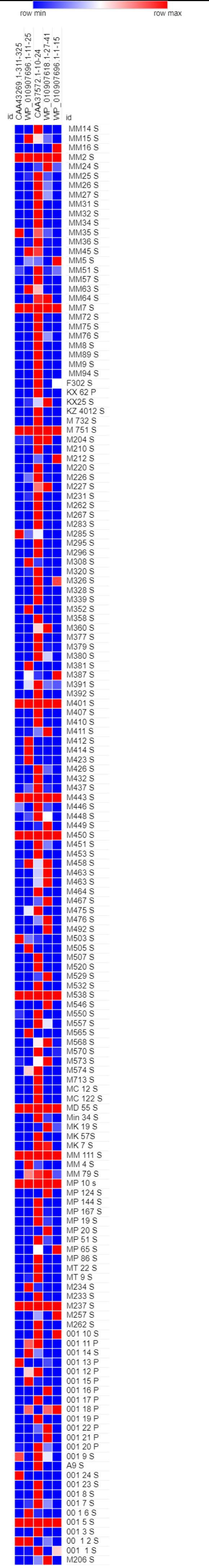

Mycobacterium leprae IgG heat map

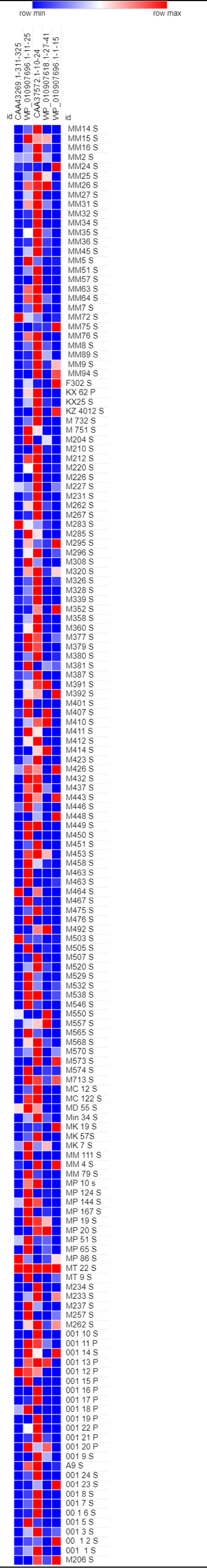

Mycobacterium leprae IgM heat map

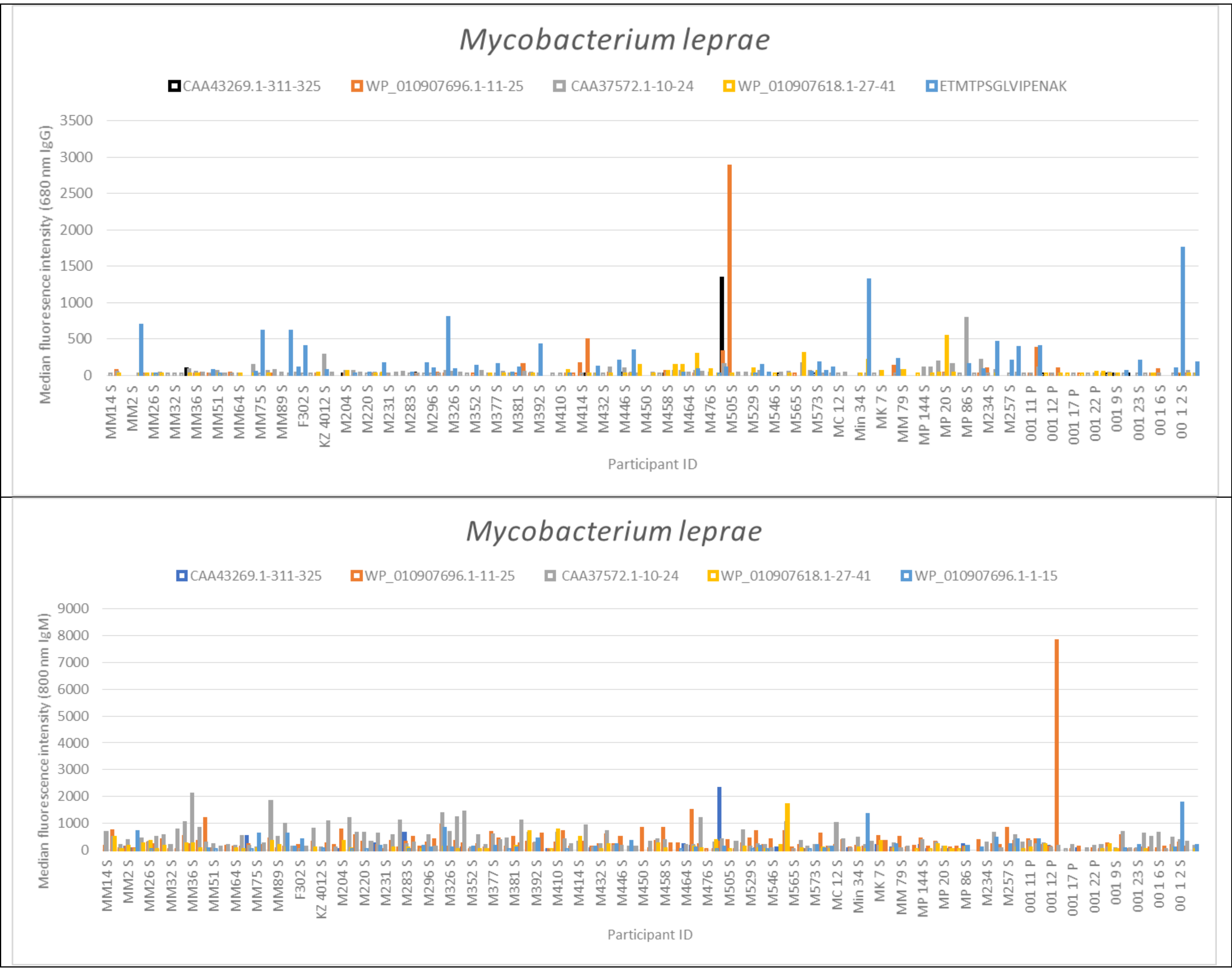

Heat maps and bar graphs generated from peptide microarray data for *Mycobacterium leprae* A. Heat maps generated from peptide microarray. Samples are arranged in rows and infection status shown on the left key. Peptides shown by their protein accession number and sequence position are arranged in columns. B. Bar graphs representing the peptide reactivity for each serum and plasma in both the infected and uninfected groups in the study.

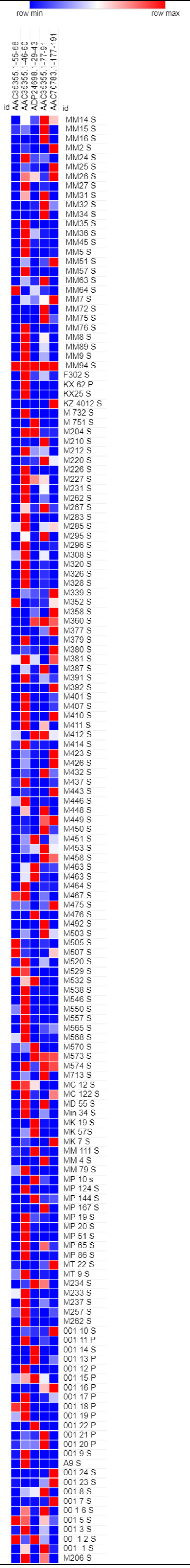

Wuchereria Bancroft IgG heat map

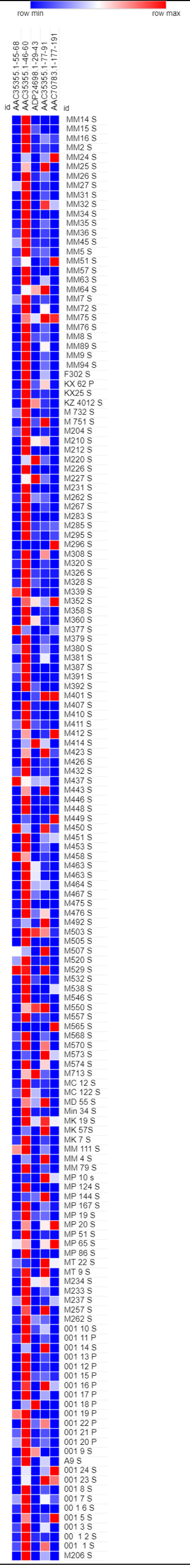

Wuchereria Bancroft IgM heat map

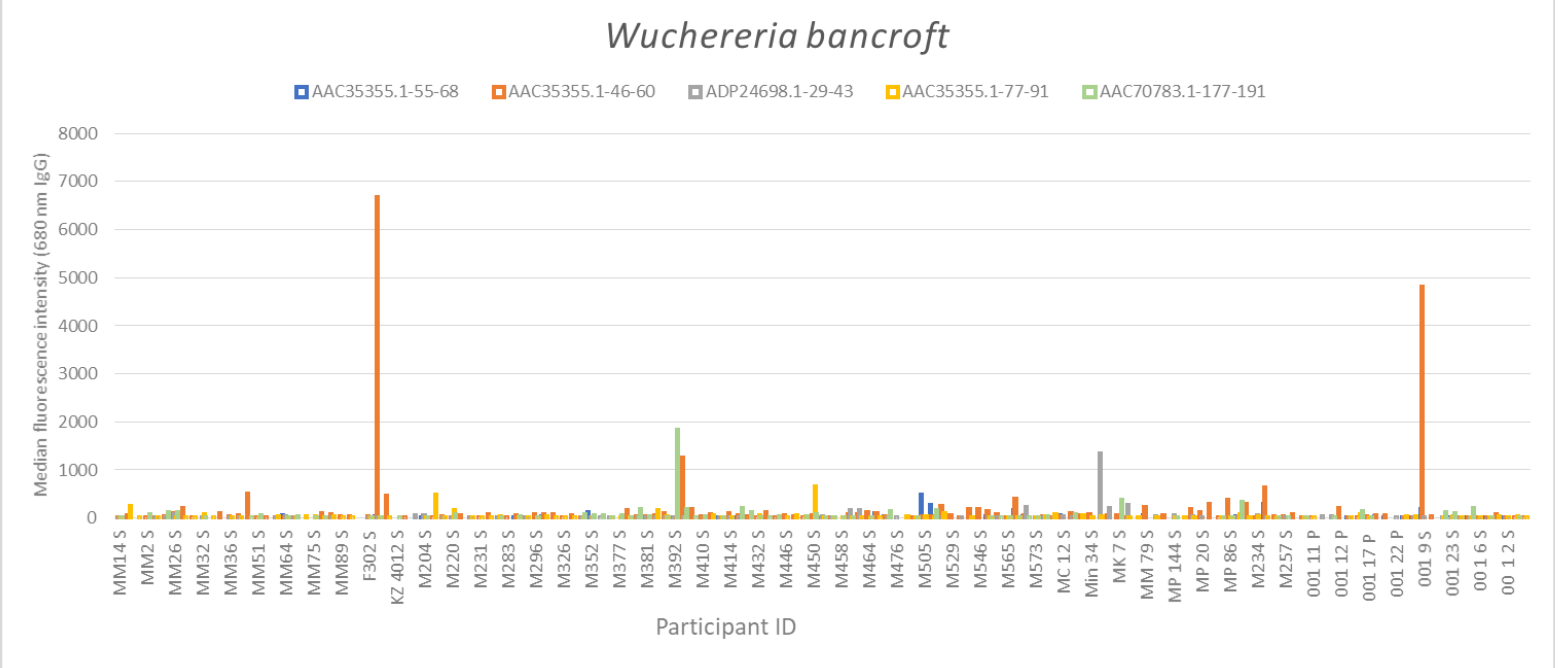

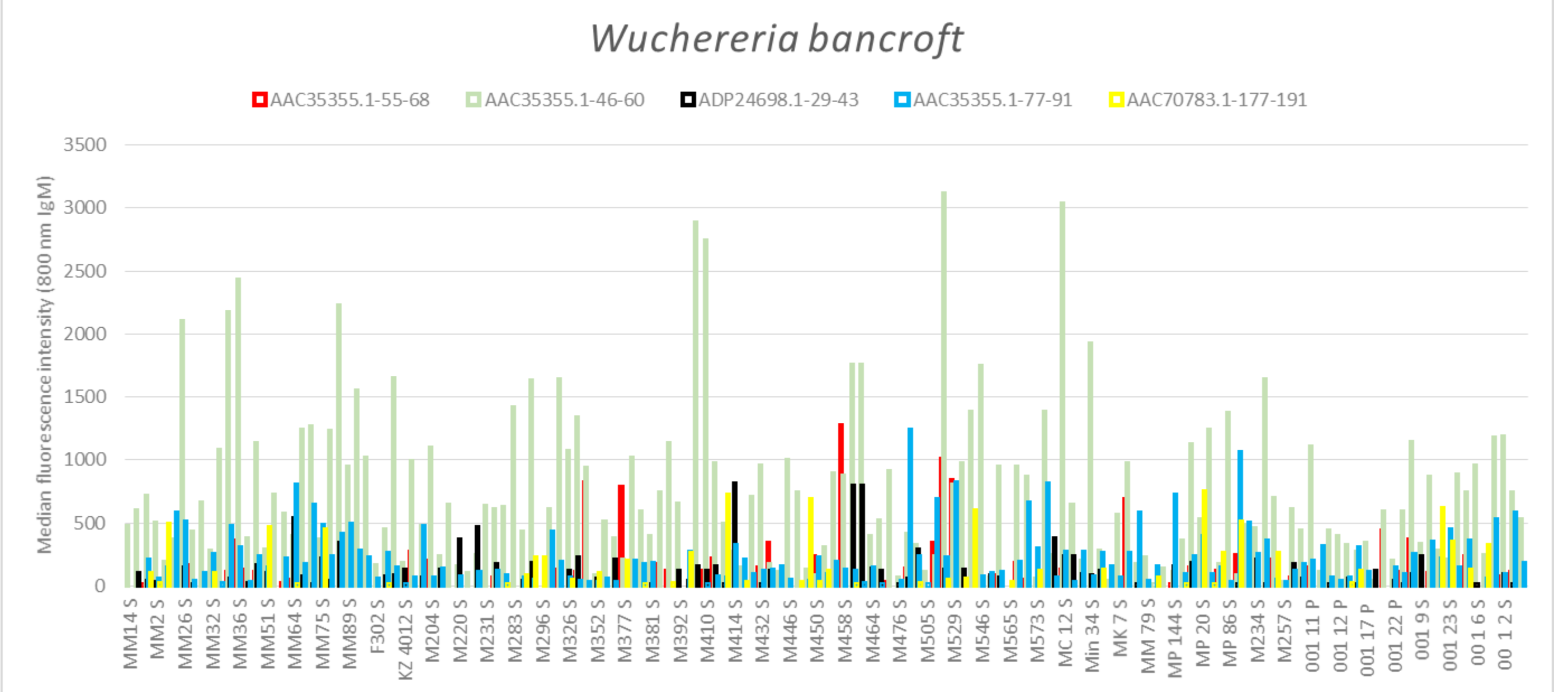

Heat maps and bar graphs generated from peptide microarray data for *Wuchereria Bancroft* A. Heat maps generated from peptide microarray. Samples are arranged in rows and infection status shown on the left key. Peptides shown by their protein accession number and sequence position are arranged in columns. B. Bar graphs representing the peptide reactivity for each serum and plasma in both the infected and uninfected groups in the study.

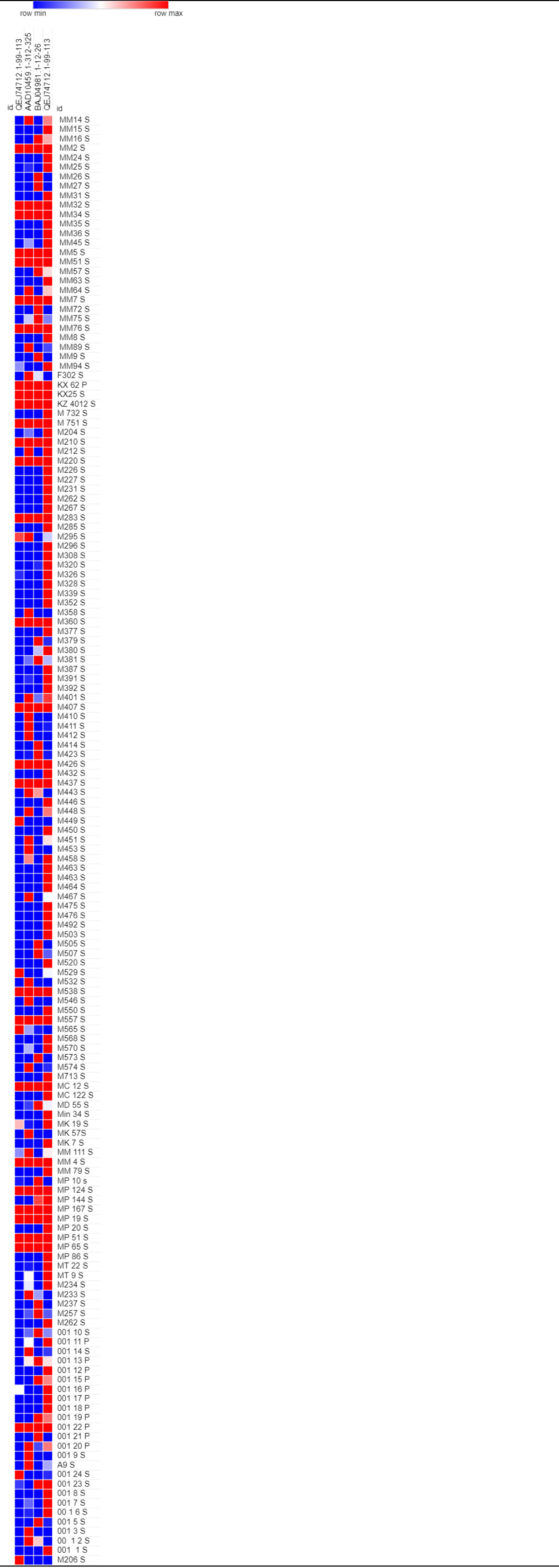

Rabies lyssavirus IgG heat map

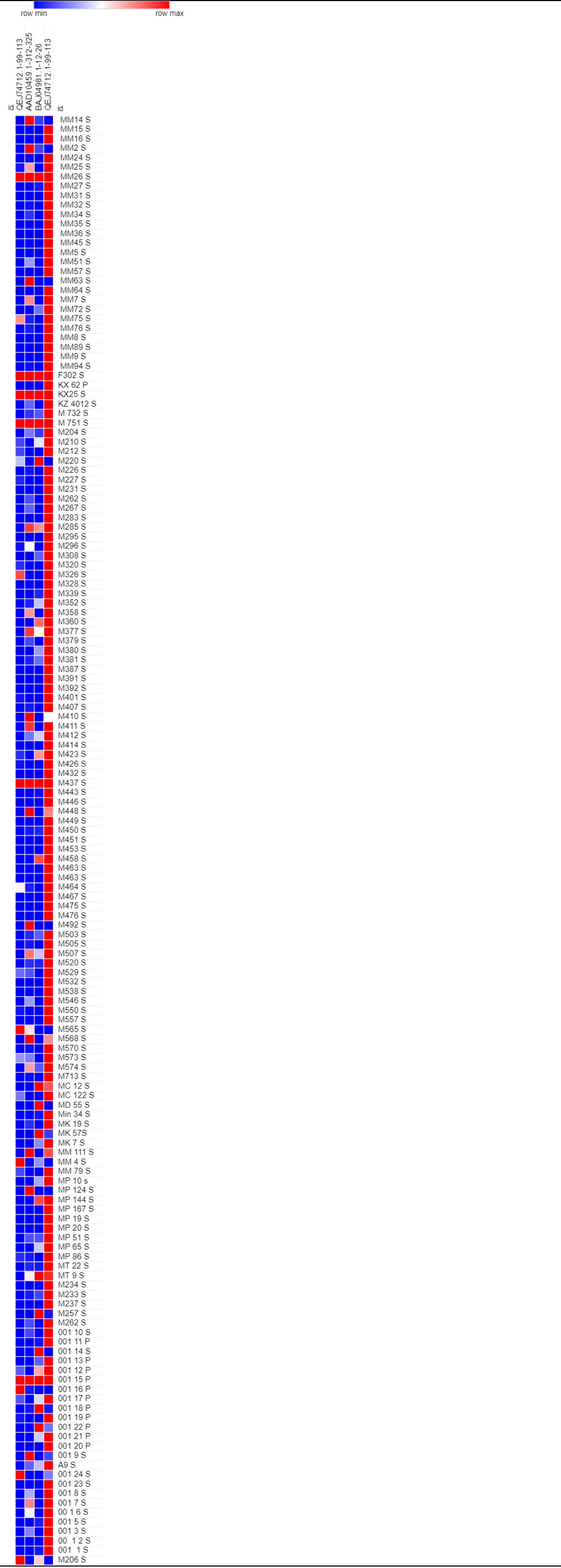

Rabies lyssavirus IgM heat map

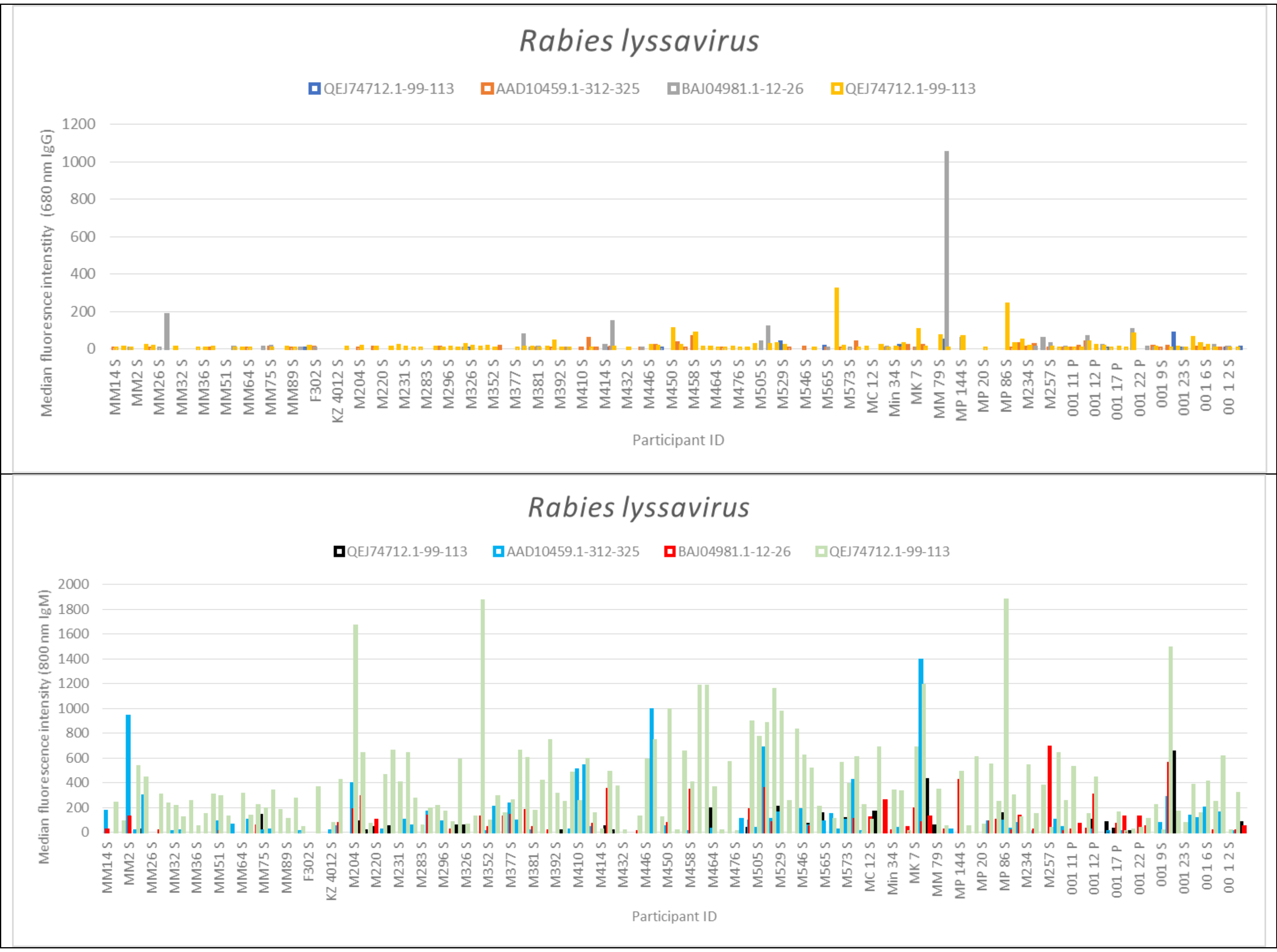

Heat maps and bar graphs generated from peptide microarray data for *Rabies lyssavirus* A. Heat maps generated from peptide microarray. Samples are arranged in rows and infection status shown on the left key. Peptides shown by their protein accession number and sequence position are arranged in columns. B. Bar graphs representing the peptide reactivity for each serum and plasma in both the infected and uninfected groups in the study.

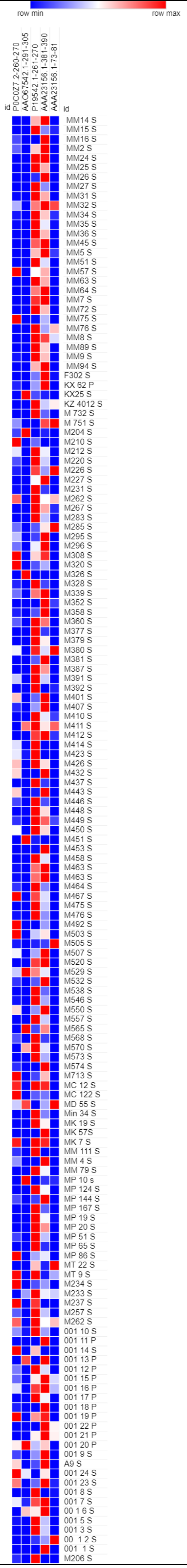

Chlamydia trachomatis IgG heat map

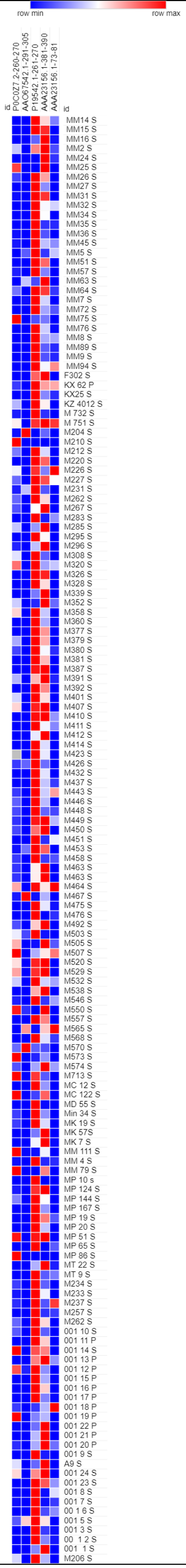

Chlamydia trachomatis IgM heat map

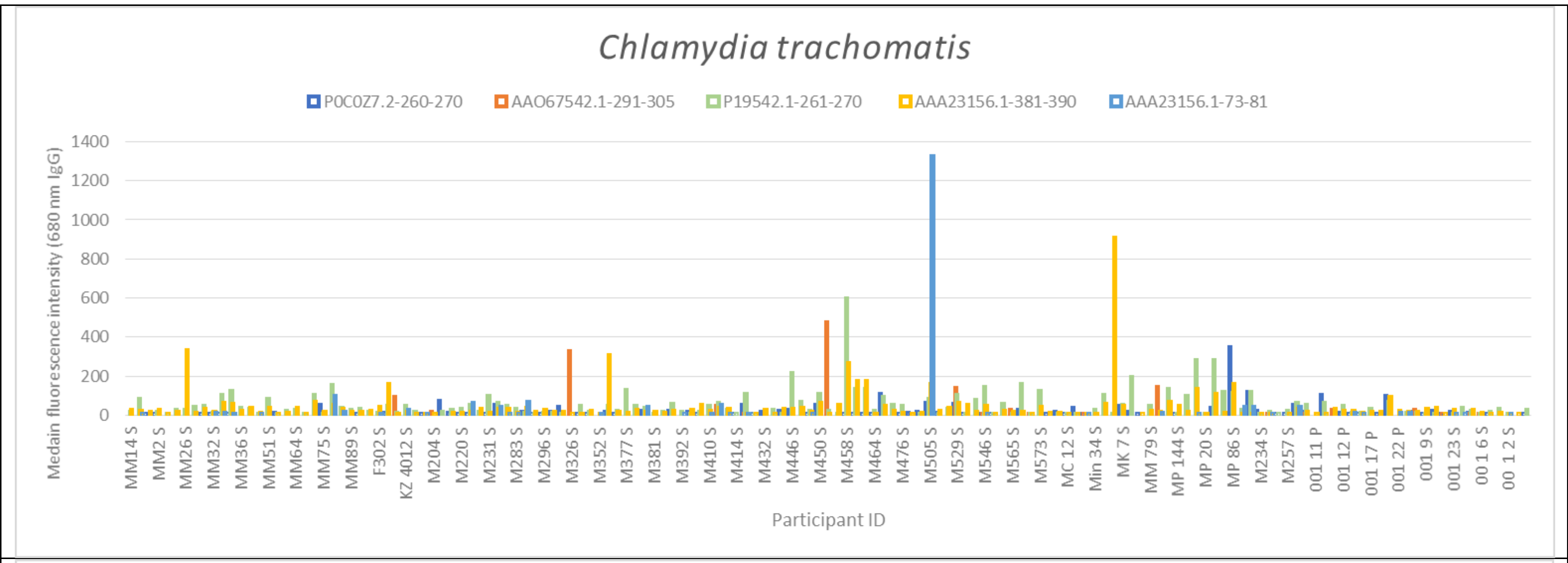

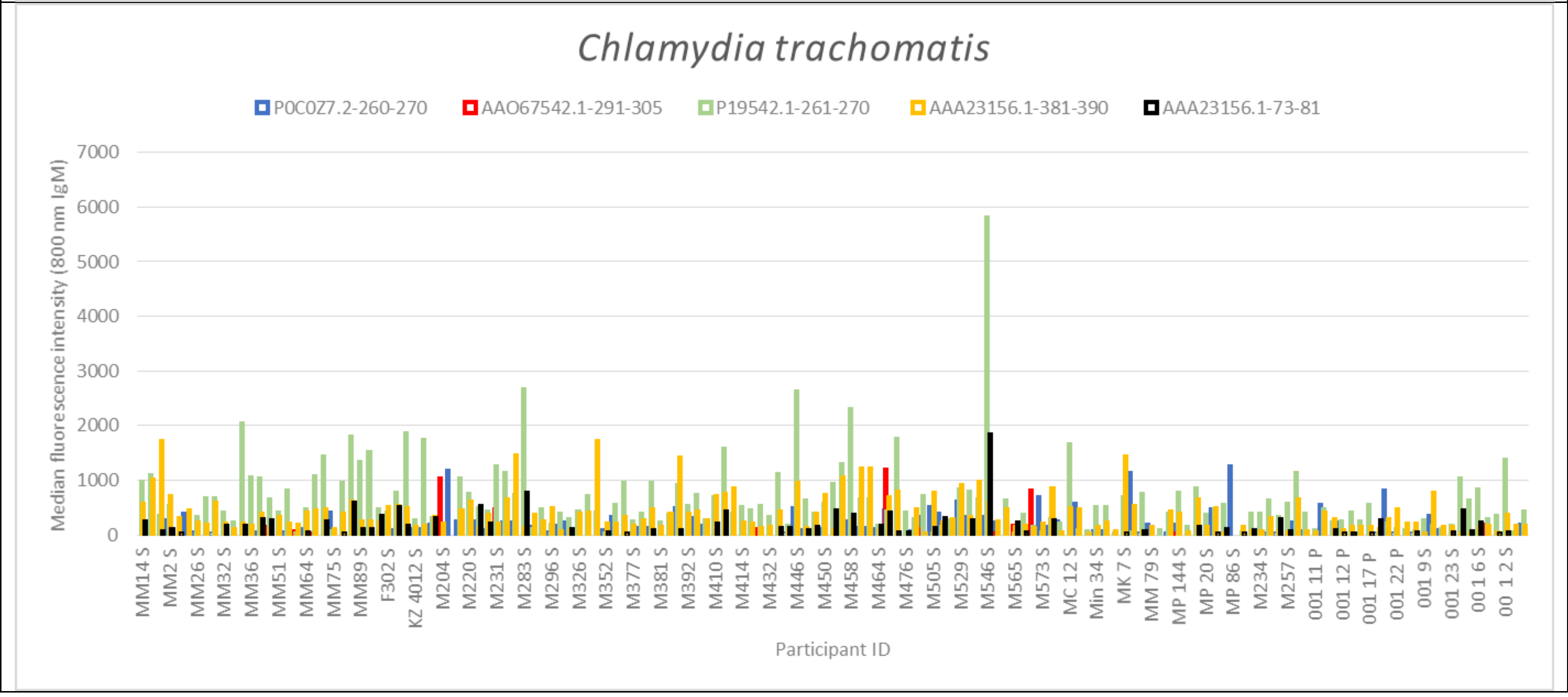

Heat maps and bar graphs generated from peptide microarray data for *Chlamydia trachomatis* A. Heat maps generated from peptide microarray. Samples are arranged in rows and infection status shown on the left key. Peptides shown by their protein accession number and sequence position are arranged in columns. B. Bar graphs representing the peptide reactivity for each serum and plasma in both the infected and uninfected groups in the study.

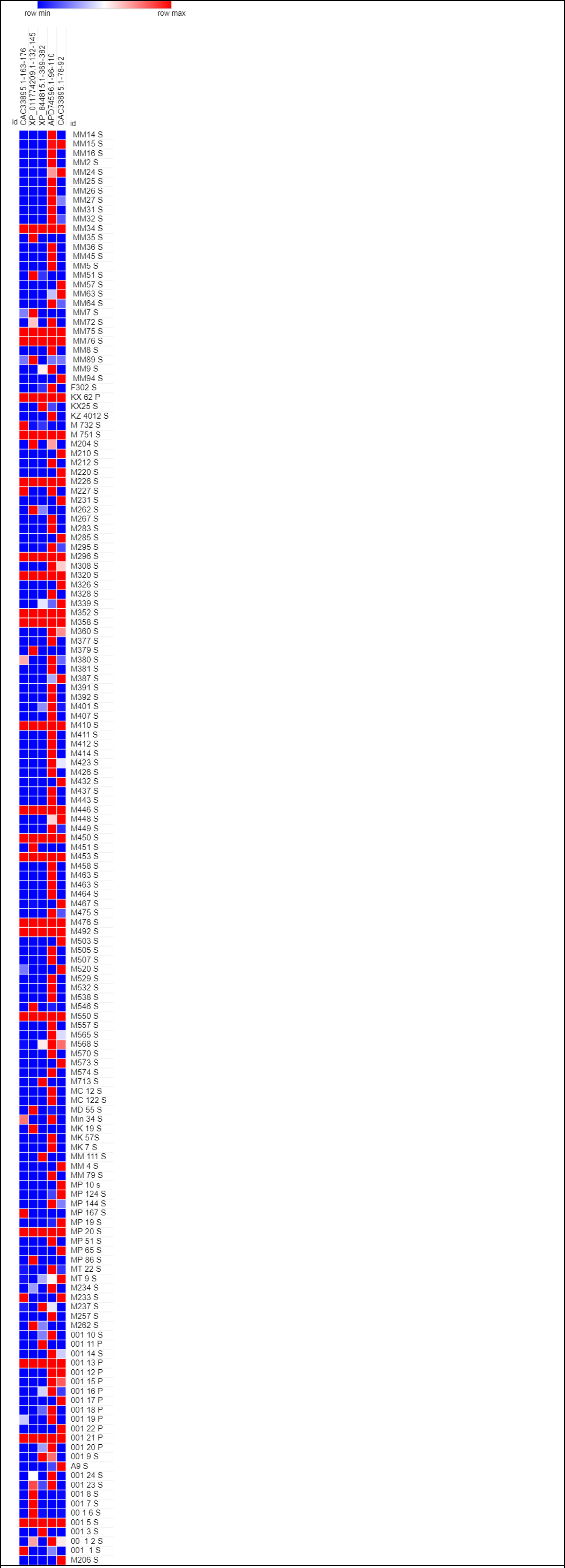

Trypanosoma brucei IgG heat map

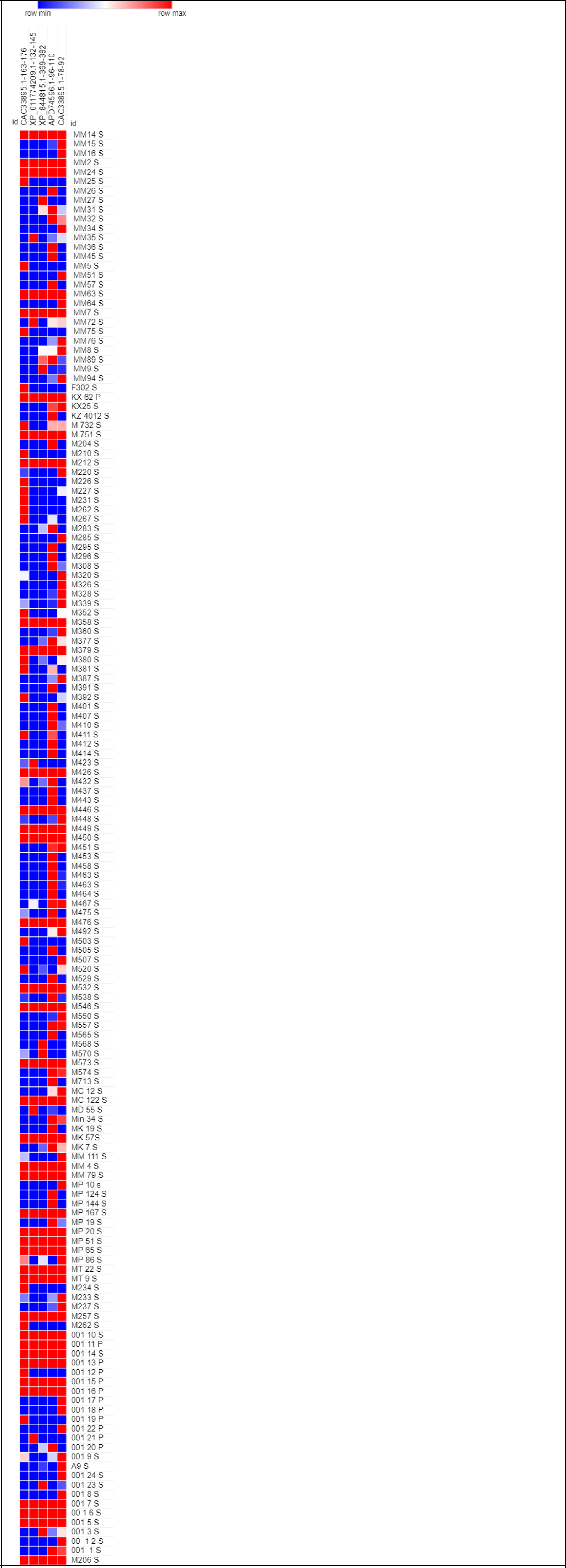

Trypanosoma brucei IgG heat map

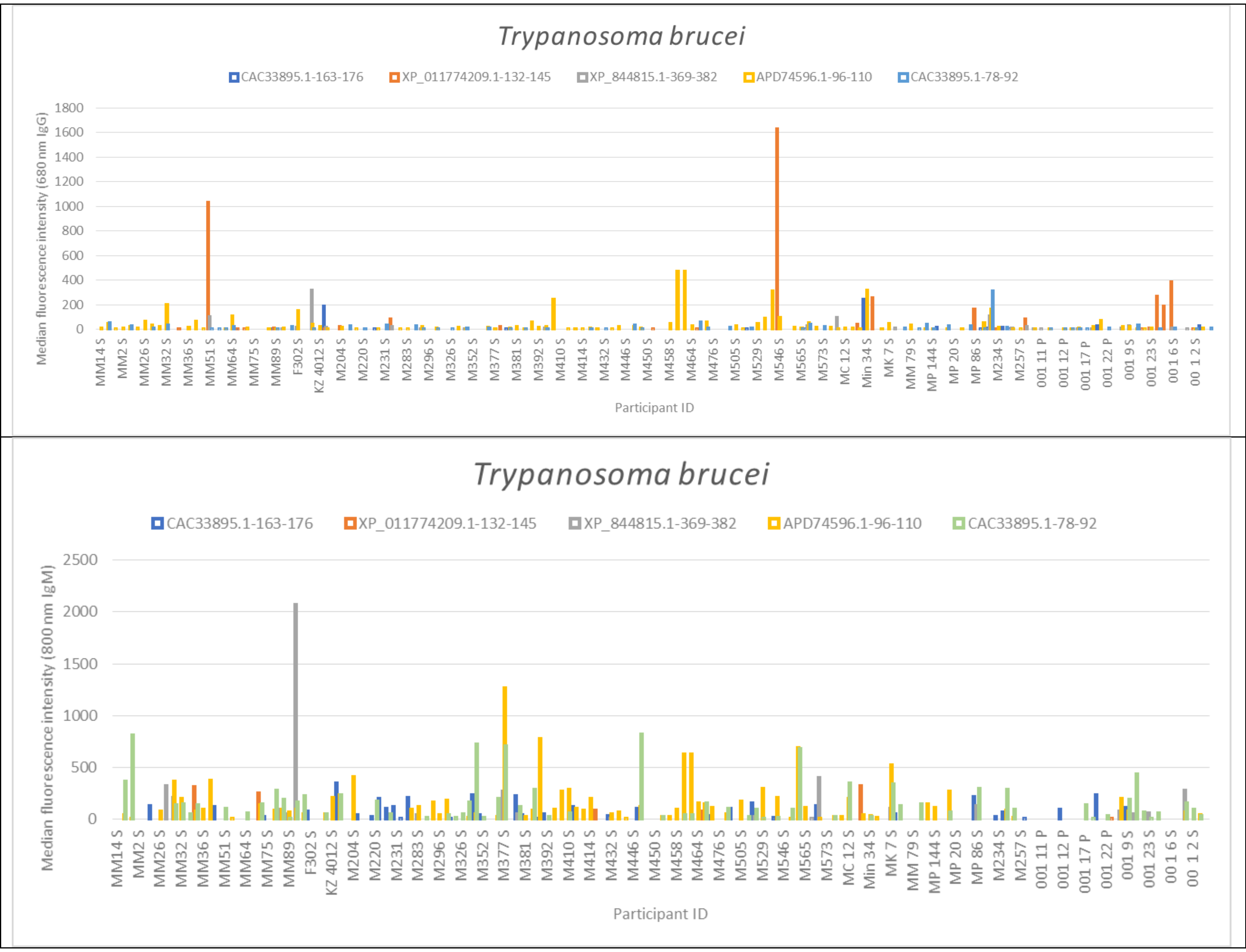

Heat maps and bar graphs generated from peptide microarray data for *Trypanosoma brucei* A. Heat maps generated from peptide microarray. Samples are arranged in rows and infection status shown on the left key. Peptides shown by their protein accession number and sequence position are arranged in columns. B. Bar graphs representing the peptide reactivity for each serum and plasma in both the infected and uninfected groups in the study.
