# Supplementary material for: Multiplex peptide microarray profiling of antibody reactivity against neglected tropical diseases derived B-cell epitopes for serodiagnosis in Zimbabwe
Source: PLoS One. 2022 Jul 22;17(7):e0271916. doi: 10.1371/journal.pone.0271916 (PMC9307155; doi:10.1371/journal.pone.0271916)
Supplement: S1 Table — (PDF) [file pone.0271916.s003.pdf]

AUC	Quality of peptide/diagnostic performance
1	Perfectly accurate test
0.9-1	Outstanding
0.8-0.9	excellent
0.7-0.8	Fair/acceptable
0.6-0.7	Poor
0.5-0.6	Fail
0	perfectly inaccurate test
